# Depressive and mania mood state detection through voice as a biomarker using machine learning

**DOI:** 10.3389/fneur.2024.1394210

**Published:** 2024-07-04

**Authors:** Jun Ji, Wentian Dong, Jiaqi Li, Jingzhu Peng, Chaonan Feng, Rujia Liu, Chuan Shi, Yantao Ma

**Affiliations:** ^1^College of Computer Science and Technology, Qingdao University, Qingdao, China; ^2^Beijing Wanling Pangu Science and Technology Ltd., Beijing, China; ^3^NHC Key Laboratory of Mental Health (Peking University), Peking University Sixth Hospital, Peking University Institute of Mental Health, National Clinical Research Center for Mental Disorders (Peking University Sixth Hospital), Beijing, China; ^4^Department of Psychology, Queen's University, Kingston, ON, Canada; ^5^School of Arts and Sciences, Brandeis University, Waltham, MA, United States

**Keywords:** voice biomarker, machine learning, mood state detection, depression, mania

## Abstract

**Introduction:**

Depressive and manic states contribute significantly to the global social burden, but objective detection tools are still lacking. This study investigates the feasibility of utilizing voice as a biomarker to detect these mood states. Methods:From real-world emotional journal voice recordings, 22 features were retrieved in this study, 21 of which showed significant differences among mood states. Additionally, we applied leave-one-subject-out strategy to train and validate four classification models: Chinese-speech-pretrain-GRU, Gate Recurrent Unit (GRU), Bi-directional Long Short-Term Memory (BiLSTM), and Linear Discriminant Analysis (LDA).

**Results:**

Our results indicated that the Chinese-speech-pretrain-GRU model performed the best, achieving sensitivities of 77.5% and 54.8% and specificities of 86.1% and 90.3% for detecting depressive and manic states, respectively, with an overall accuracy of 80.2%.

**Discussion:**

These findings show that machine learning can reliably differentiate between depressive and manic mood states via voice analysis, allowing for a more objective and precise approach to mood disorder assessment.

## 1 Introduction

The World Health Organization (WHO) estimates that 40 million people had bipolar disorder (BPD) and 280 million people suffered from depression in 2019 (1). Depression, sometimes referred to as unipolar depression or major depressive disorder (MDD), is a mental illness that affects a person’s everyday functioning and is characterized by recurrently low emotions or a loss of interest in activities. It might be challenging to differentiate bipolar illness from depression because of its repeated episodes of mania/hypomania and depression (2). In China, the lifetime prevalence rates of bipolar disorder and depression are 0.6% and 6.8%, respectively (3), and the prevalence is still rising as a result of COVID-19 (4). Mental disorder can increase the risk of suicide. People with depression are 20 times more likely to commit suicide (5). Depression has become the fourth leading cause of death (6). Patients with manic episodes tend to exhibit elevated mood, a tendency to anger easily, and excessive sensitivity. Over time, these conditions can lead to physical fatigue and compromised immune systems. Furthermore, they may engage in high-risk activities due to impulsiveness, posing potential harm to themselves and society.

At present, the detection of depression and manic mood are usually made by observer-based clinical rating scales, such as the Mood Disorder Questionnaire (MDQ), Quick Inventory of Depressive Symptomatology (QIDS), and Young Mania Rating Scale (YMRS). These scales are used as gold standards to evaluate the severity of manic and depressive symptoms, which are based on the Diagnostic and Statistical Manual of Mental Disorders, Fifth Edition (DSM-V) (7). Certainly, the precision of these rating scales depends on participant compliance and the subjective interpretation of practitioners. Therefore, developing continuous, objective assessments of symptom severity would be groundbreaking.

In recent years, many machine learning and deep learning methods are used to automatic mood recognition by speech. Faurholt-Jepsen et al. (8) presented voice analysis as an objective state marker in bipolar disorder in 2016, however the study only included the speech features of 28 participants. Shin et al. (9) suggested a speech biomarker machine learning model for the identification of moderate and serious depression in 2021. Lin et al. (10) proposed a deep learning method for diagnosing depressive orders; Punithavathi et al. (11) conducted an empirical investigation that demonstrated the potential of machine learning-based voice recognition techniques for depression prediction; and Shen et al. (12) proposed a GRU/BiLSTM-based model for depression detection. The advantages and disadvantages of these studies are shown in Table 1.

**Table 1:** Previous studies and their advantages and disadvantages.

References	Authors	Advantage	Disadvantage
(8)	Faurholt-Jepsen et al.	We are the first to use speech analysis as an objective biomarker for bipolar disorder. 2. An objective and non-invasive diagnostic method has been proposed.	The relatively small number of participants (28 people) may limit the generalization ability of the study.
(9)	Shin et al.	A machine learning model of voice biomarkers is proposed for identifying moderate and severe depression. It may provide a new approach for early identification and intervention of depression.	The model accuracy is relatively low, with sensitivity and specificity of only 0.65 and 0.66.
(10)	Lin et al.	Using deep learning methods for the diagnosis of depressive disorders may improve accuracy. Deep learning models may be able to process complex voice data.	The amount of data in the remission period is relatively small, making it difficult to verify statistical differences. There is noise in the speech environment, and the noise reduction is insufficient.
(11)	Punithavathi et al.	An empirical study demonstrates the potential of machine learning-based speech recognition technology in predicting depression. It provides new possibilities for early identification and intervention of depression.	The model accuracy needs to be improved.
(12)	Shen et al.	A GRU/BiLSTM-based model is proposed for depression detection, which may improve the accuracy of diagnosis. The GRU/BiLSTM structure is able to handle long-term dependencies in sequential data.	Depression detection methods, not the depressive mood.

The Transformer model has gained popularity in recent years, first appearing in textual analysis to handle long-range dependencies in text. Since its introduction by Vaswani et al. (13), the model has made great progress in both speech and text recognition. Zhang and colleagues (14) presented a hybrid model for depression detection that combines Transformers and BiLSTM. Moreover, BERT is a pre-trained Transformer model for text analysis that was introduced by Devlin et al. (15). Using Wav2Vec 2.0 (16) that has already been trained, Banno et al. (17) devised a method for assessing oral proficiency in English. Guo et al. (18) developed a pre-trained model appropriate for Chinese in 2022. Therefore, in voice recognition, pre-trained models are now commonly employed, providing novel methods for recognizing emotions.

While speech is a useful tool for categorizing depression and manic episodes in these models, their drawbacks include a lack of characteristics and insufficient examination of the transitions between these states. This study suggests three models to differentiate between depression, mania, and remission emotions in order to address these constraints. It also contrasts these models to find out if using pre-trained models improves classification accuracy. This study developed a voice-based machine learning model for diagnosing mania and depression by analyzing 1,337 voice messages from 93 participants. We suggest that voice can function as an objective marker for supplementary diagnosis of emotional states by the analysis of objective speech data, allowing for the predictive forecasting of emotional phase transitions.

## 2 Materials and methods

### 2.1 Data sample

A self-monitoring app named MoodMirror was loaded onto the participants’ smartphones, and an alarm was set to sound once a day at a time of their choosing to remind the patients to provide electronic self-monitored data. The participants completed all assessment questionnaires, verified their informed consent, and obtained trial information via the app.

In this current longitudinal investigation, the following hypotheses were tested using the MoodMirror system in participants presenting with moderate to severe degrees of manic and depressed symptoms: In naturalistic settings, voice features from ordinary life that were extracted using the “mood diary” module may distinguish between different affective states. Participants are asked to describe their present naturalistic mood state in the “mood diary” module stated above. Participants’ smartphones recorded audio at a sample rate of 16 kHz.

Every participant was chosen from Peking University Sixth Hospital and provided written informed consent electronically via the MoodMirror app. Based on the subjects’ current depressive and manic states, a sample of 1,337 voice messages from 93 subjects was divided into three groups: the depression mood state group (431 voice messages from *n* = 39), the mania mood state group (208 voice messages from *n* = 20), and the remission group (698 voice messages from *n* = 34). The demographic information of the participants is shown in Table 2.

**Table 2:** Comparing the demographics based on various emotional states.

		Depressive	Manic	Remission	*F/χ^2^*	*p*-value
N		39 (41.94%)	20 (21.51%)	34 (36.56%)		
Gender	Female	19 (48.72%)	11 (55.00%)	27 (79.42%)	6.86	0.032
	Male	19 (48.72%)	8 (40.00%)	7 (20.58%)		
	Absence	1 (2.56%)	1 (5.00%)	0 (0%)		
Age (years)		29.74 ± 12.04	29.68 ± 8.03	42.05 ± 9.14	13.15	<0.001
Education (years)		14.32 ± 2.69	14.06 ± 3.45	17.84 ± 1.87	17.46	<0.001
Marriage	Married	5 (7.69%)	4 (20.00%)	24 (70.59%)	32.77	<0.001
	Unmarried	31 (79.49%)	15 (75.00%)	8 (23.53%)		
	Divorced	2 (5.13%)	0 (0.00%)	0 (0.00%)		
	Absence	1 (2.56)	1 (5.00%)	2 (5.88%)		

### 2.2 Clinical assessments

The MoodMirror app incorporates three self-evaluated scales that are considered gold standards for assessing manic and depressed mood states: the Mood Disorder Questionnaire (MDQ) (19), the Quick Inventory of Depressed Symptomatology (QIDS) (20) and the Young Mania Rating Scale (YMRS) (21). Concurrently, the voice recordings were gathered within the application. Table 3 displays the various combinations of cut-offs that were used to determine the remission, depressive, and manic mood states. Table 4 provides an illustration of the detailed scare scores. ANOVA test was used to compare score differences among three groups.

**Table 3:** Standard of many emotional states.

Mood state	Criterion
Remission	MDQ < 7 and YMRS < 13 and QIDS < 9
Depressive	MDQ < 7 and YMRS < 13 and QIDS ≥ 9
Manic	(MDQ ≥ 7 or YMRS ≥ 13) and QIDS ≥ 9

**Table 4:** Clinical traits according to various emotional states.

		Depressive	Manic	Remission	*F*	*p*-value
*N*		39	20	34		
MDQ	Mean	1.25	9.00	1.27	208.44	<0.001
	SD	1.58	2.67	1.51		
QIDS	Mean	17.25	22.18	2.89	153.59	<0.001
	SD	5.82	6.97	2.04		
YMRS	Mean	2.77	13.59	1.67	58.57	<0.001
	SD	3.79	11.62	2.92		

MDQ, Mood Disorder Questionnaire; QIDS, Quick Inventory of Depressive Symptomatology; YMRS, Young Mania Rating Scale.

### 2.3 Voice features

In this study, we used a total of 22 voice features: 8 time-domain features (zero-crossing rate, short-term energy, short-term energy entropy, spectral centroid, spectral spread, spectral entropy, spectral flux, spectral rolloff), 13 Mel-Frequency Cepstral Coefficients (MFCC) features, and the duration of the voice recording. Speech contains rhythm and tempo, and longer segments provide more audio information. Thus, feature extraction from longer speech segments captures crucial information more precisely. Longer speeches allow for more fine-grained segments during short-time framing, leading to more accurate information extraction. Consequently, speech duration impacts speech classification, and we included it as a feature in our analysis. The specific meanings of each feature are presented in Table 5.

**Table 5:** Conception of features.

ID	Feature	Conception
1	Zero-crossing rate	The number of times a signal changes from positive to negative or from negative to positive within a unit of time
2	Short-term energy	The magnitude of sound energy within a certain period of time
3	The short-term energy entropy	The energy distribution characteristics of speech signals in the time domain, which is obtained by calculating the information entropy of short-term energy
4	Spectral centroid	The central position of the spectral distribution of an audio signal, the location where the spectral energy is concentrated
5	Spectral spread	The distribution of an audio signal around its spectral center, as well as the dispersion degree of spectral energy
6	Spectral entropy	The relationship between the power spectrum and entropy rate of an audio signal, which can be used to describe the complexity and randomness of the signal
7	Spectral flux	The rate of spectral changes in an audio signal between adjacent time frames, reflecting the dynamic characteristics of the audio signal
8	Spectral rolloff	The rate of spectral attenuation in an audio signal, measuring the degree of attenuation in the audio signal
9–21	Mel-Frequency Cepstral Coefficients(MFCC)	It is primarily employed to convert audio signals into compact and information-rich representations for tasks such as speech recognition and speaker identification (22)
22	Duration	The duration of a speech utterance

In general, a smaller value of spectral centroid indicated that the spectral energy of the audio signal was more concentrated in the low-frequency range. A greater spectral spread indicated a wider distribution of spectral energy across the frequency domain in the audio signal. Spectral rolloff represented the frequency that was below a specified percentage of the total spectral energy. Mel-Frequency Cepstral Coefficients (MFCC) was a commonly used feature extraction technique in speech and audio signal processing. By extracting MFCCs, audio signals were transformed into a compact set of feature vectors, which could be more easily utilized for tasks such as classification, recognition, or other tasks using machine learning algorithms.

The above-mentioned features id1–id21 were extracted from each frame, then, the average of all frame features was calculated to serve as the feature representation of the speech. The duration of speech was also the feature. Utilizing χ^2^ tests for detecting differences between groups, we selected features with significant differences to train the model. Table 6 shows the inter-group comparison of features for the three categories. The violin plots representing the distributions of three groups of speech features are shown in Supplementary File 1. Features were extracted using the open-source program pyAudioAnalysis (23) of Python version 3.6.1.

**Table 6:** Differences in features of three types of speech.

Feature	Depressive	Manic	Remission	*χ^2^*	*p*-value
Zero-crossing rate	0.130 ± 0.057	0.120 ± 0.048	0.105 ± 0.039	101.993	<0.001
Short-term energy	0.020 ± 0.032	0.025 ± 0.031	0.013 ± 0.023	93.421	<0.001
The short-term energy entropy	3.024 ± 0.220	2.935 ± 0.197	2.977 ± 0.079	246.895	<0.001
Spectral centroid	0.229 ± 0.067	0.214 ± 0.060	0.196 ± 0.045	138.894	<0.001
Spectral spread	0.221 ± 0.029	0.207 ± 0.030	0.206 ± 0.017	183.481	<0.001
Spectral entropy	1.055 ± 0.560	0.905 ± 0.475	0.856 ± 0.373	52.667	<0.001
Spectral flux	0.015 ± 0.012	0.017 ± 0.009	0.014 ± 0.006	60.850	<0.001
Spectral rolloff	0.231 ± 0.155	0.210 ± 0.135	0.175 ± 0.107	67.486	<0.001
MFCC1	−29.834 ± 4.72	−28.578 ± 2.697	−28.361 ± 2.49	108.089	<0.001
MFCC2	1.886 ± 0.671	2.027 ± 0.520	2.185 ± 0.417	72.105	<0.001
MFCC3	−0.074 ± 0.359	0.123 ± 0.545	0.183 ± 0.213	149.193	<0.001
MFCC4	0.225 ± 0.287	0.176 ± 0.317	0.339 ± 0.165	77.060	<0.001
MFCC5	−0.011 ± 0.210	−0.016 ± 0.208	0.213 ± 0.186	329.659	<0.001
MFCC6	0.064 ± 0.149	0.054 ± 0.157	0.067 ± 0.125	2.786	0.248
MFCC7	−0.055 ± 0.151	0.034 ± 0.169	0.042 ± 0.089	165.106	<0.001
MFCC8	−0.085 ± 0.146	0.020 ± 0.109	−0.002 ± 0.087	123.477	<0.001
MFCC9	−0.039 ± 0.119	0.003 ± 0.150	−0.014 ± 0.081	17.099	<0.001
MFCC10	−0.099 ± 0.134	−0.045 ± 0.123	−0.217 ± 0.091	379.390	<0.001
MFCC11	−0.106 ± 0.115	−0.015 ± 0.138	−0.047 ± 0.071	111.395	<0.001
MFCC12	−0.035 ± 0.115	0.012 ± 0.124	−0.015 ± 0.061	39.043	<0.001
MFCC13	−0.064 ± 0.104	−0.054 ± 0.110	−0.106 ± 0.063	90.403	<0.001
Duration	45.005 ± 28.981	10.371 ± 10.989	32.720 ± 15.234	212.314	<0.001

### 2.4 Machine learning models

After the feature extraction process from the original speech recordings, leave-one-subject-out was used validation method. The Gate Recurrent Unit (GRU) (12) is a gating mechanism in recurrent neural networks (24), which is similar to LSTM (long short-term memory) (25) with an output gate and fewer parameters. GRU is included in this study since it performs similarly to LSTM with fewer parameters on tasks including speech signal modeling and natural language processing (26, 27).

Bidirectional Long Short-Term Memory (BiLSTM) (28) is a further development of LSTM and BiLSTM combines the forward hidden layer and the backward hidden layer, which can access both the preceding and succeeding information (28). The application of BiLSTM in speech processing typically involves leveraging its bidirectional recurrent structure to capture temporal information in speech signals, thereby improving performance in tasks such as speech recognition and speech emotion recognition.

**Figure 1:**
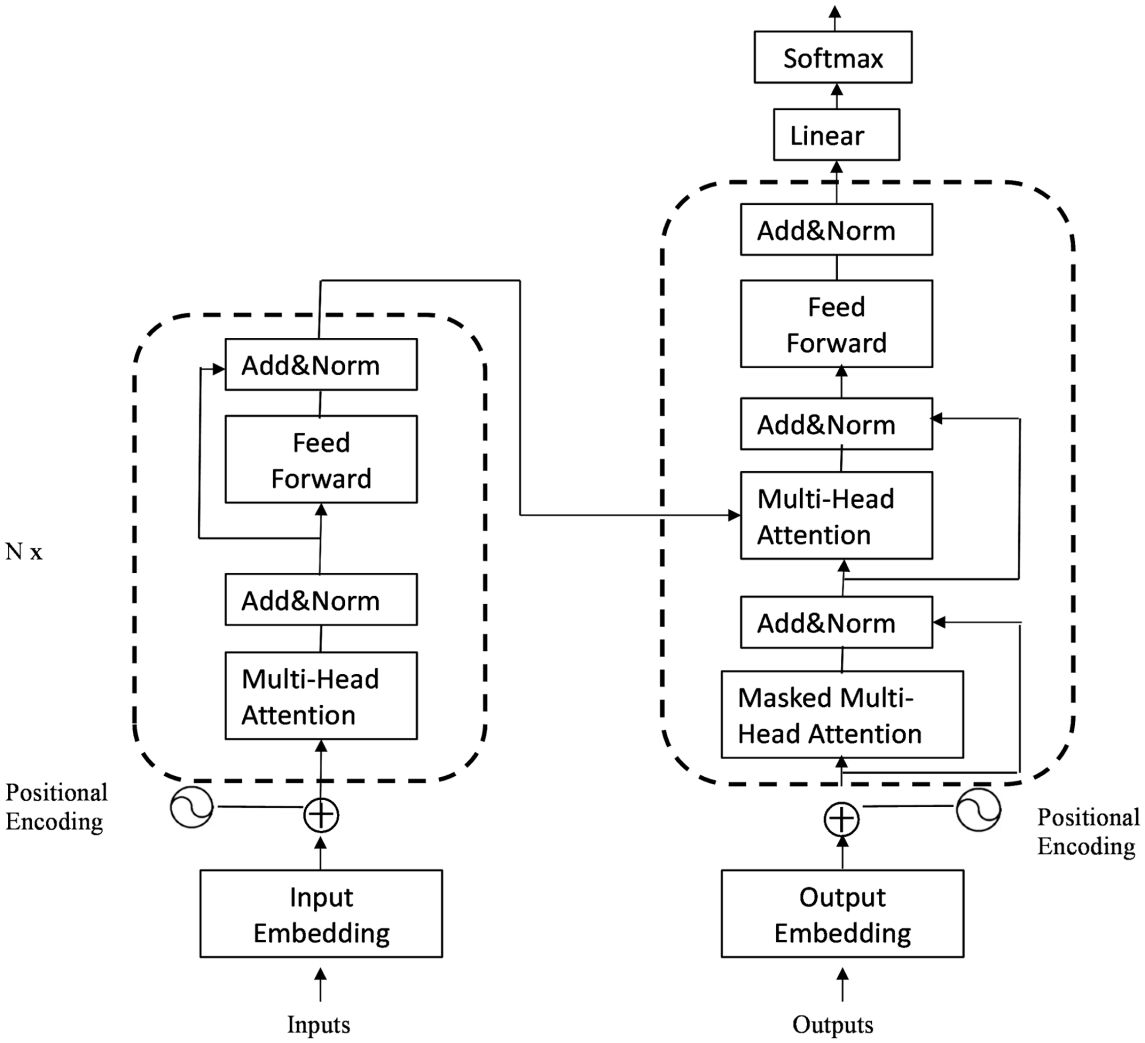
The structure of transformer.

Wav2vec2.0 (16) is a speech feature extraction model proposed by the Facebook AI Research team, which utilizes Transformer as its underlying architecture. The structure of transformers is illustrated in Figure 1. The Chinese-speech-pretrain model used in this study is developed by TencentGames and Tencent Zhiji based on Wav2vec2.0, which has been trained using a large amount of unsupervised learning data. These data primarily originate from YouTube and Podcast, covering a wide range of recording scenarios, background noises and speaking styles. The model was trained using the Fairseq toolkit (29) and employed the 10,000 h Chinese dataset from the train_1 set of WenetSpeech (30) as the foundation. This self-supervised learning approach enables the model to learn the deep structure of speech without annotations, enhancing its comprehension of Chinese speech.

Linear Discriminant Analysis (LDA) is a linear learning algorithm originally proposed by Fisher (31) in the field of classification. For binary classification problems, LDA projects both categories of data onto a single line to ensure that data within the same category are as close as possible, while data from different categories are as far apart as possible, which means minimizing the intra-class distance and maximizing the inter-class distance. When a new sample is encountered, it is also mapped onto this line, and the category of the new sample is determined based on the position of its projection point (32).

### 2.5 Processing

First of all, voice recordings and questionnaire data (QIDS, MDQ, and YMRS scores) were collected from 93 patients. The questionnaire responses were organized according to Table 3 criteria to classify patients into depressive, manic, or remission states based on their mood. To mitigate environmental noise variability in self-recorded voices, a band-pass filter (200–4,000 Hz) was applied to improve the signal-to-noise ratio (SNR). The Kruskal-Wallis test was used to compare SNR differences among the three speech groups, while the Mann–Whitney U test was employed to analyze differences between any two speech types. Furthermore, an endpoint detection method based on short-time energy and zero-crossing rate was implemented to remove silent intervals at the beginning and end of each speech sample. Subsequently, speech signals were segmented into frames with a frame duration of 30 ms and a 15 ms frame shift. Each frame underwent Hamming windowing to prepare for subsequent feature extraction.

Initially, the 93 participants were numbered from 1 to 93, and voice features were extracted for each individual. The leave-one-subject-out cross-validation method was employed for model validation. Specifically, the voice data of participant ID1 served as the test set, while the data from the remaining 92 participants constituted the training set. To address class imbalance, the Synthetic Minority Over-sampling Technique (SMOTE) was applied during training data preparation. For evaluating the models trained using Linear Discriminant Analysis (LDA), Gate Recurrent Unit (GRU), and Bi-directional Long Short-Term Memory (BiLSTM), the voice data from participant ID1 was input into each trained model. The accuracy, specificity, and sensitivity of these models in recognizing voice emotions were computed accordingly. Subsequently, participant ID2’s voice data was used as the test set, and the process of training the models with the data from the remaining 92 participants was repeated. This cycle was iterated until each of the 93 participants had served as the test set at least once, allowing for a comprehensive evaluation of the trained models. Finally, the average performance across all 93 test iterations was calculated to determine the overall effectiveness of the models.

In addition, default parameters were used in LDA. And for GRU and BiLSTM, grid search was employed to determine the optimal parameters. The parameters to be determined include batch-size, with options of 30, 40, 50; dropout rate with options of 0.1, 0.2, 0.3; and learning rate with options of 0.006, 0.0006, 0.00006. Both GRU and BiLSTM use ReLU and Softmax as the activation functions for the fully connected layers. The aforementioned training process was implemented using PyTorch (33). As a result, the batch-size, dropout and learning rate of GRU were 40, 0.3 and 0.0006, respectively, while for BiLSTM, the batch-size, dropout and learning rate were 40, 0.2 and 0.0006, respectively.

This study retrieved features using the pre-trained model Wav2vec2.0, and the best results were obtained by training using the aforementioned ideal parameters and model.

## 3 Results

Table 2 presents demographic information. Gender, marital status, years of schooling, and age showed significant differences (*p* < 0.05) across the three groups.

Table 4 illustrates that there are differences among the three groups of people in the three scales of MDQ, QIDS and YMRS (*p* < 0.05).

The average signal-to-noise ratio (SNR) for depressive mood speech is 5.838 ± 3.211, while the average SNR for manic mood speech is 5.586 ± 3.211, and the SNR for the speech during remission period is 4.561 ± 2.281. Significant differences in SNR were found among these groups (K = 143.954, *p* < 0.05). Specifically, there was no significant difference in SNR between depressive and manic mood speech (U = 42,872, *p* = 0.379). However, SNR differed significantly between the remission period and depressive mood speech (U = 101,271, *p* < 0.05), as well as between the remission period and manic mood speech (U = 38,077, *p* < 0.05).

**Figure 2:**
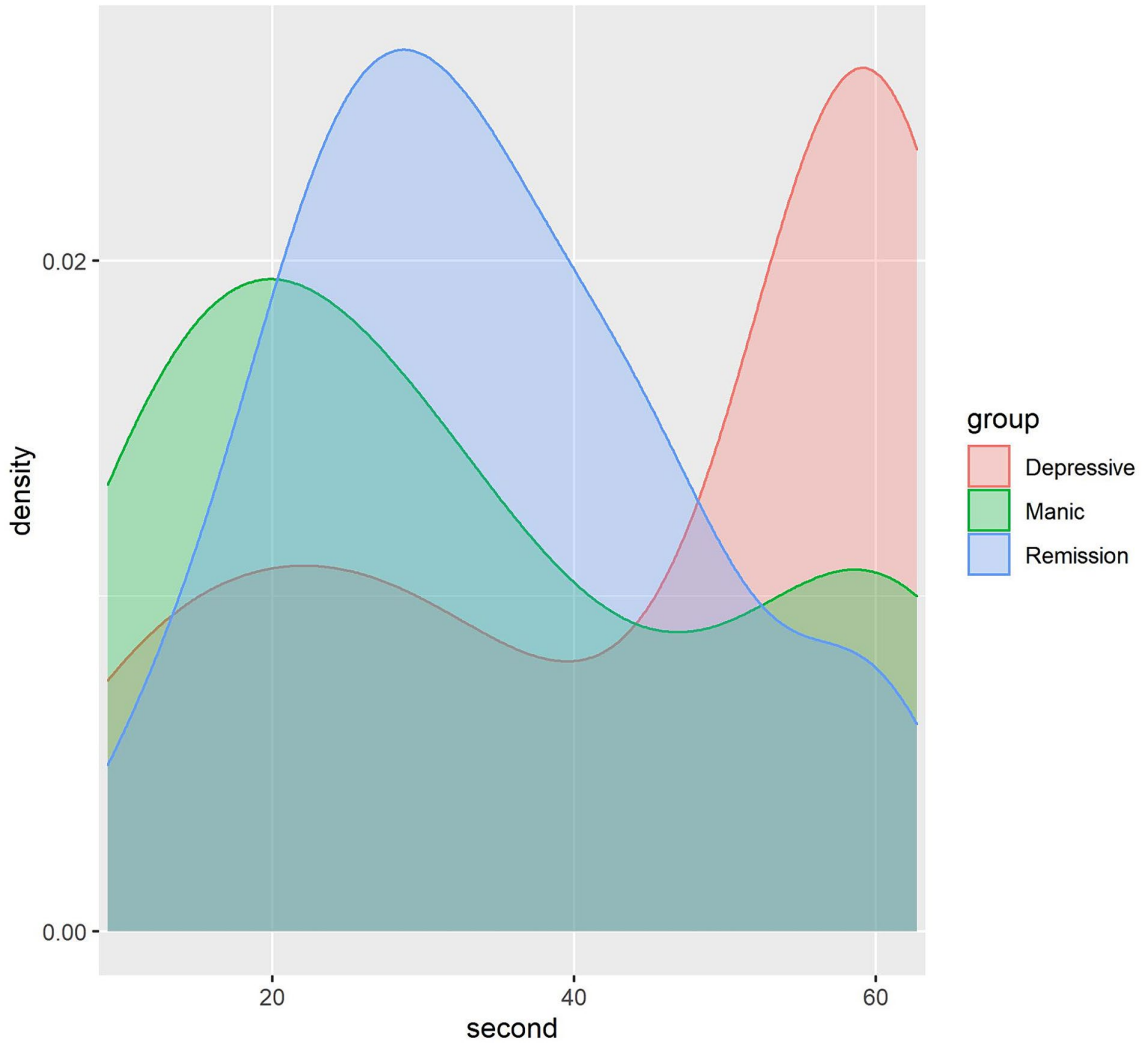
Density map showing the dispersion of voice recording duration.

As is shown in Figure 2, the speech length of people in the depressive mood state is concentrated in 40–60 s, the speech length of the manic mood state is concentrated in 1–30 s, and the speech length of the people in the remission is concentrated in 20–40 s. The speech duration typically ranges from 1 to 60 s, with graphical representation continuing beyond 60 s. This extension occurs due to a peak near the 60-s mark, where the graph automatically extends around the peak instead of abruptly stopping. The duration of speech varies with different emotions, which correlates with emotional states. Individuals experiencing depression often communicate via electronic devices like mobile phones, showing a strong inclination to express themselves (34, 35). They may exhibit slower speech due to lower mood and energy levels, along with reduced interaction with others. This slower articulation could stem from delayed thought processes. Conversely, individuals in a manic state tend to speak rapidly, reflecting impatience and haste, resulting in shorter recorded speech durations.

**Figure 3:**
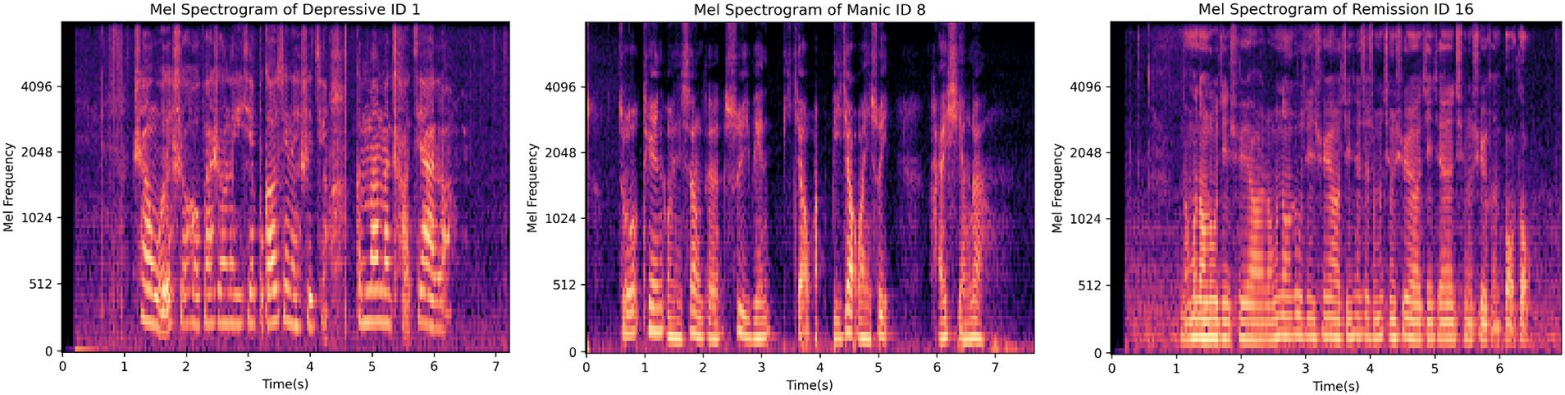
Participants’ Mel spectrograms showing depressive, manic and remission mood states.

Moreover, Figure 3 shows participants’ Mel spectrograms depressive, manic and remission mood states. In a Mel spectrogram, time is depicted on the horizontal axis and frequency on the vertical axis, with brighter areas indicating higher energy levels. The figure illustrates that the sound energy across the three different emotional states is primarily concentrated in lower frequencies. Specifically, the depressive state and remission period exhibit concentration below 2,048 Hz, while the manic mood is concentrated below 1,024 Hz. A comparative analysis reveals that compared to the remission period, the energy distribution in the depressive state is relatively more dispersed, while in the remission period, energy is denser than 512 Hz. This observation highlights discernible distinctions in energy and frequency among the three emotional states, thereby facilitating the discrimination of speech emotions. As can be seen in Figure 3, the MFCC plots of the depressive and the remission are similar, while the manic mood state is significantly different from them. This might be attributed to depression and mania being two extremes of emotion, thus potentially exhibiting starkly different patterns in speech features.

Table 6 demonstrates the contrasting differences of 22 features among depressive mood, manic mood, and remission mood voices. It is evident from Table 6 that, except for the MFCC6 feature, which showed no significant difference among the three voice types (*p* = 0.248), the remaining 21 features exhibited significant differences (*p* < 0.001). Supplementary File 1 presents violin plots of the 21 features excluding MFCC6, which indicate variations in the distributions of these 21 features across the three types of voice. For detailed information, please refer to Supplementary File 1. These 21 features are the selected ones for model training.

Lastly, by using the selected 21 features to train the model, the confusion matrix and performance of the model is shown in Tables 7, 8. The Chinese-speech-pretrain-GRU achieves an accuracy of 80.2%, outperforming LDA (78.9%), BiLSTM (69.5%), and GRU (70.0%). BiLSTM demonstrates 82.2% specificity and 56.4% sensitivity for detecting depressive mood, and 85.3% specificity and 46.2% sensitivity for manic mood. 60.1% precision and 58.2% F1-score for detecting depressive mood, and 36.6% precision and 40.8% F1-score for manic. Similarly, GRU exhibits 57.3% sensitivity, 82.4% specificity, 60.8% precision and 59.0% F1-score for depressive mood, and 56.3% sensitivity, 86.2% specificity, 42.8% precision and 48.6% F1-score for manic mood.

**Table 7:** The confusion matrix of LDA, BiLSTM, GRU and Chinese-speech-pretrain+GRU.

		Predicted			Predicted		
		Remission	Depressive	Manic	Remission	Depressive	Manic
		LDA			BiLSTM		
True label	Remission	622	44	32	593	58	47
	Depressive	44	303	84	69	243	119
	Manic	30	48	130	9	103	96
		**GRU**			**Chinese-speech-pretrain + GRU**		
True label	Remission	572	89	37	625	39	34
	Depressive	65	247	119	22	334	75
	Manic	21	70	117	7	87	114

LDA, Linear Discriminant Analysis; BiLSTM, Bidirectional Long Short-Term Memory; GRU, The Gate Recurrent Unit.

**Table 8:** Performance comparison between LDA, BiLSTM, GRU, and Chinese-speech-pretrain+GRU.

	Remission	Depressive	Manic	Remission	Depressive	Manic
	LDA			BiLSTM		
Accuracy	0.789			0.695		
Sensitivity	0.891	0.703	0.625	0.849	0.564	0.462
Specificity	0.884	0.898	0.897	0.878	0.822	0.853
Precision	0.894	0.767	0.528	0.884	0.601	0.366
F1-score	0.892	0.734	0.572	0.866	0.582	0.408
	**GRU**			**Chinese-speech-pretrain + GRU**		
Accuracy	0.700			0.802		
Sensitivity	0.819	0.573	0.563	0.896	0.775	0.548
Specificity	0.865	0.824	0.862	0.955	0.861	0.903
Precision	0.869	0.608	0.428	0.925	0.750	0.529
F1-score	0.843	0.590	0.486	0.956	0.726	0.511

Further, for depressed conditions, the Chinese-speech-pretrain-GRU shows 77.5% sensitivity, 86.1% specificity, 75.0% precision and 72.6% F1-score, and for manic states, 54.8% sensitivity, 90.3% specificity, 52.9% precision and 51.1% F1-score. LDA, in contrast, reaches 62.5% sensitivity and 89.7% specificity, 52.8% precision and 57.2% F1-score for manic states and 70.3% sensitivity, 89.8% specificity, 76.7% precision and 73.4% F1-score for depressed conditions. LDA performs better than BiLSTM and GRU, but Chinese-speech-pretrain-GRU performs better overall. Table 7 shows the confusion matrix of four model. Table 8 presents the specific findings.

## 4 Discussion

This study shows that Chinese-speech-pretrain-GRU, BiLSTM, LDA and GRU can be utilized for voice analysis of mood state detection, and the Chinese-speech-pretrain-GRU can distinguish depressive and manic mood with 80.2% accuracy, the LDA can distinguish depressive and manic mood with 78.9% accuracy, the GRU can distinguish depressive and manic mood with 70%, while the BiLSTM with 69.5% accuracy. It means machine learning models can distinguish those mood states through objective speech, and pre-train models can further extract the information embedded in the speech, thus improving prediction accuracy. Furthermore, speech can serve as a biomarker to differentiate between depressive and manic moods.

In recent years, several scholars have explored multimodal models for identifying depressive and manic emotions. Ye et al. (36) introduced a hybrid model integrating voice and text for depression detection. Zheng et al. (37) proposed a multitask model capable of simultaneous emotion recognition and depression detection. Alghowinem et al. (38) developed a machine learning-based multimodal depression detector, showing significant advancements over unimodal approaches. Future studies could involve not only voice but also text, video, and image modalities. This extension may provide a thorough comprehension of differences between manic, depressed, and remission phases from many angles, improving the precision of emotion identification.

Patients with bipolar disorder have the characteristics of “polarization” of emotions. The methods and focuses of interventions for manic and depressive episodes are different. During manic episodes, it is necessary to prevent the harm caused by mania. However, during a depressive episode, we need to pay attention to the risk of self-injury and suicide, and consider using antidepressants in combination (39). By integrating a model that can effectively identify different phases from mobile devices such as mobile phones, and having patients or their family members regularly upload their voices, remote and regular monitoring of the emotional phases of bipolar patients can be achieved, so that intervention measures and interventions for patients can be adjusted in a timely manner. The treatment plan provides a feasible solution to achieve personalized treatment of bipolar disorder and save the human and material costs required for mental illness management. This study also has certain auxiliary value in the diagnosis of bipolar disorder. Because patients are lack of knowledge for themselves disease and may be in a depressive episode when seeking treatment, bipolar disorder is easily misdiagnosed as unipolar depression. The ideas proposed in this study can help doctors discover missed manic phases in clinical practice.

When the sound wave in the voice is affected by aerodynamic factors and generates mechanical vibration, it is converted into a sound source signal to generate (40). The voice contains biological acoustic characteristics such as spectrum, prosody, and formant (41, 42). The length of the voice has an impact on the amount and accuracy of the features contained in the voice. Vogel and Morgan studied that the length of voice data would affect the accuracy of biological characteristics in the voice (43). Scherer et al. showed through research that the accuracy of disturbance measurement in the voice was affected by the duration of the voice, and only voice over 3 s could provide accurate features (44). There are also studies proving that the pitch measurement of long voice is more accurate than that of short voice (45, 46). To conclude, in this study, we observed that patients with different emotional states exhibited varying voice durations, indicating that the information carried in their voices differs. Significant differences in voice durations were found among patients in depressive, manic, and remission states. However, we did not study the impact of voice duration on the internal characteristics of speech. Future research will further analyze the specific impact of duration on these speech characteristics.

This study also has certain limitations. Firstly, the number of participants included in this study is limited, and the use of self-reported scales may lead to inaccuracies of the emotion labels. Secondly, regarding the quantity of speech data, the number of recordings varies among patients, which may introduce bias to the model. Thirdly, in the study, we only used voice as a biomarker to investigate the recognition of emotions. Therefore, in order to enhance the accuracy and generalizability of the results, the future study will increase the number of participants, control the quantity of speech samples per patient, and utilize alternative techniques to ascertain the patients’ emotional states. In subsequent research, apart from speech, we will introduce information such as images, videos and texts to detect depressive mood, manic mood and remission emotion through a Multi-Modal machine learning approach.

## Data availability statement

The raw data supporting the conclusions of this article will be made available by the authors, without undue reservation.

## Ethics statement

The studies involving humans were approved by the Medical Ethics Committee of Peking University Sixth Hospital (Institute of Mental Health). The studies were conducted in accordance with the local legislation and institutional requirements. The participants provided their written informed consent to participate in this study.

## Author contributions

JJ: Writing – original draft. WD: Conceptualization, Supervision, Writing – review & editing. JL: Methodology, Writing – original draft. JP: Methodology, Writing – original draft. CF: Data curation, Writing – review & editing. RL: Data curation, Writing – review & editing. CS: Writing – review & editing. YM: Conceptualization, Funding acquisition, Writing – review & editing.
