# Supplementary material for: Depressive and mania mood state detection through voice as a biomarker using machine learning
Source: Front Neurol. 2024 Jul 4;15:1394210. doi: 10.3389/fneur.2024.1394210 (PMC11254794; doi:10.3389/fneur.2024.1394210)
Supplement: Supplementary file 1 [file Data_Sheet_1.PDF]

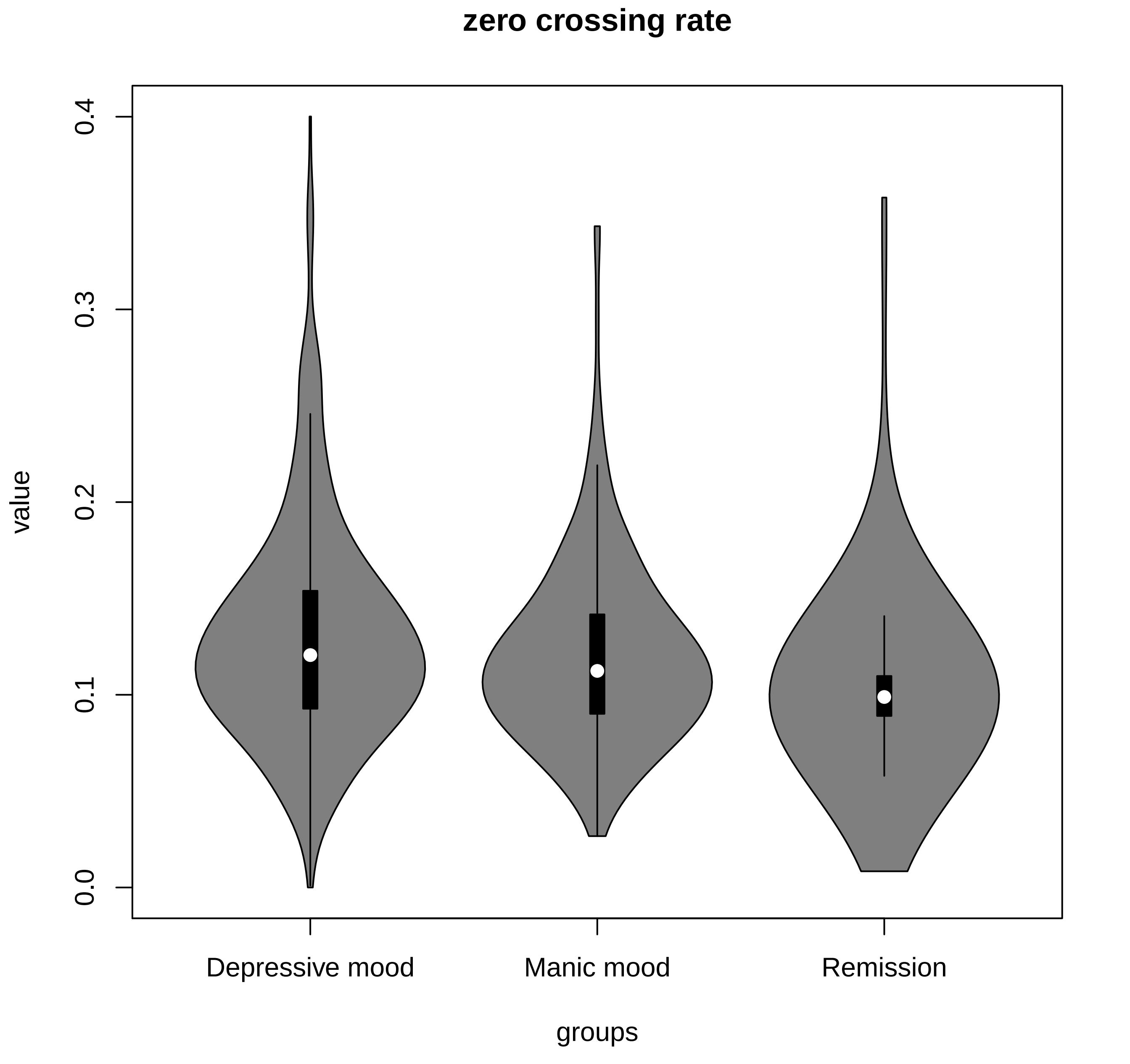

zero crossing rate
value
0.0
0.1
0.2
0.3
0.4
Depressive mood
Manic mood
Remission
groups

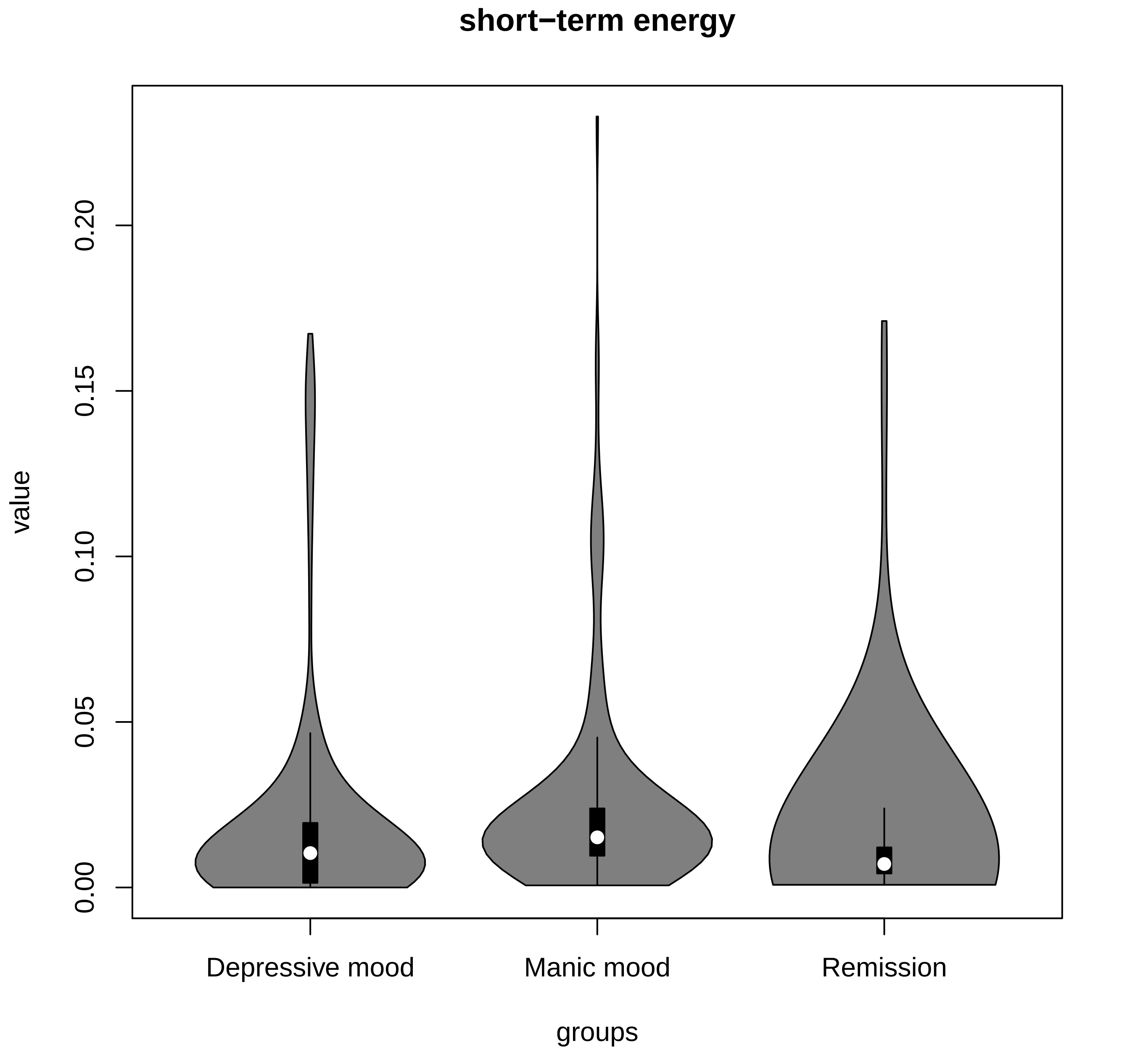
short−term energy
value
0.00
0.05
0.10
0.15
0.20
Depressive mood
Manic mood
Remission
groups

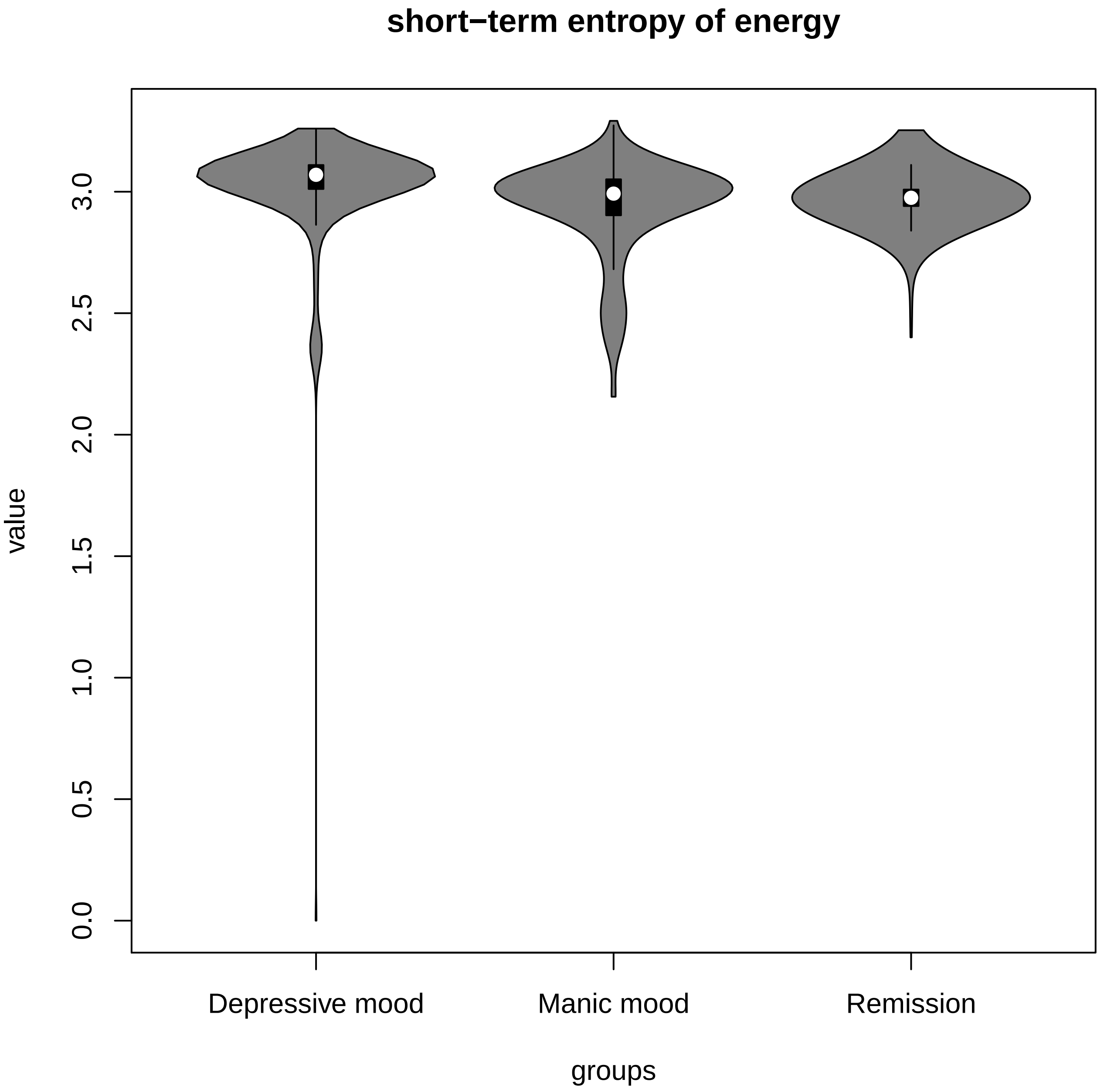
short−term entropy of energy
value
0.0
0.5
1.0
1.5
2.0
2.5
3.0
Depressive mood
Manic mood
Remission
groups

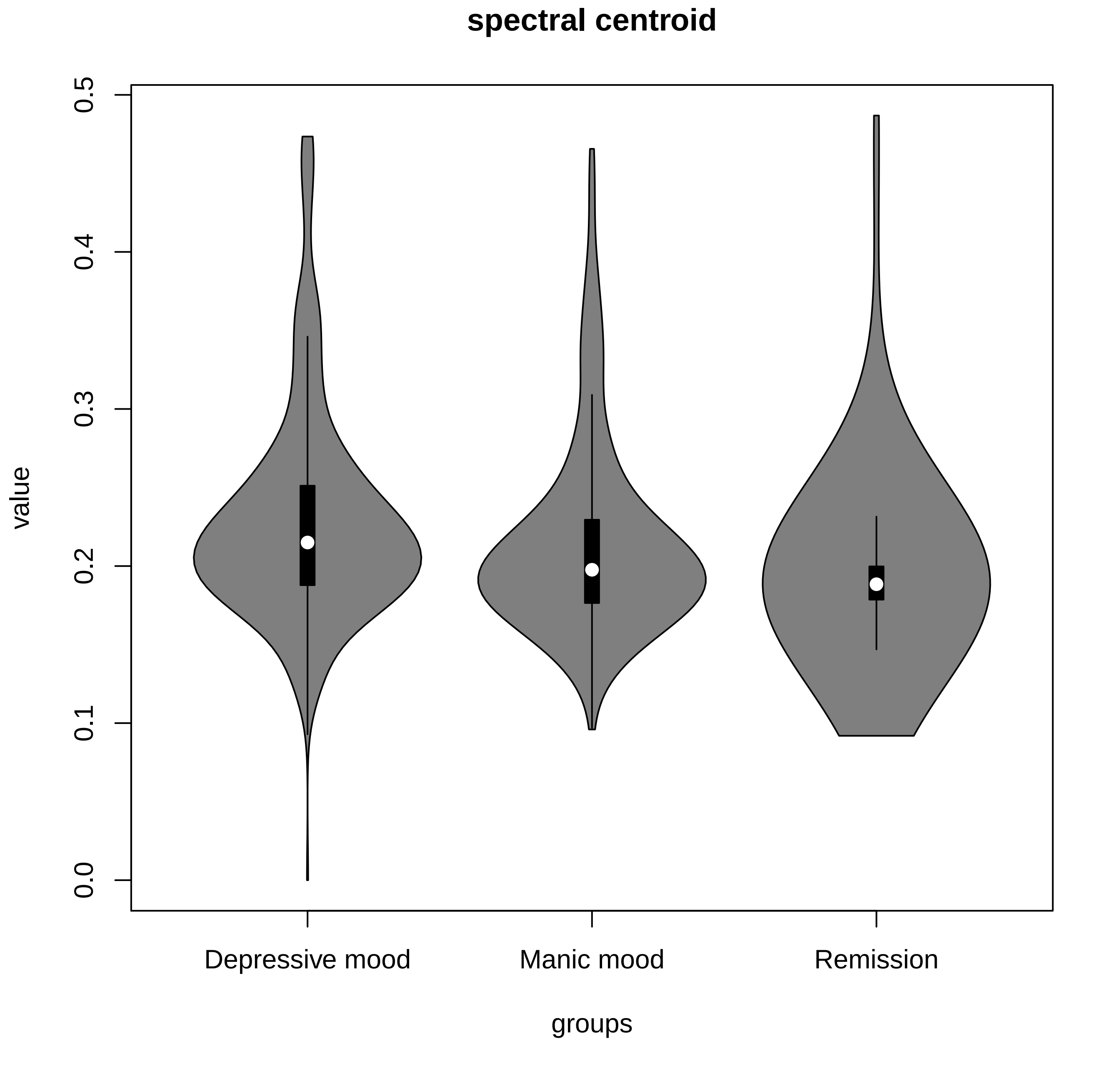
spectral centroid
value
0.0
0.1
0.2
0.3
0.4
0.5
Depressive mood
Manic mood
Remission
groups

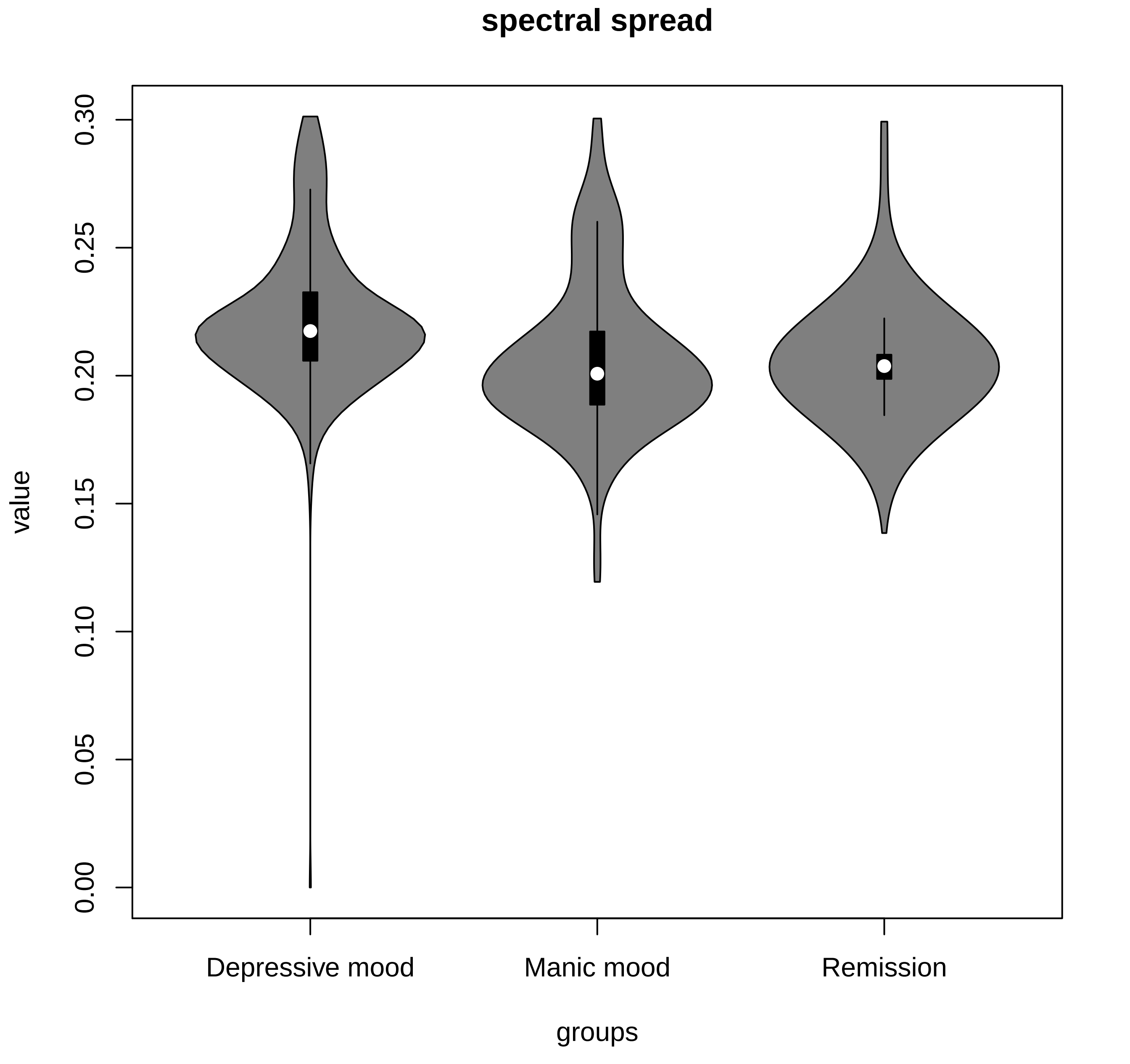
spectral spread
value
0.30
0.25
0.20
0.15
0.10
0.05
0.00
Depressive mood
Manic mood
Remission
groups

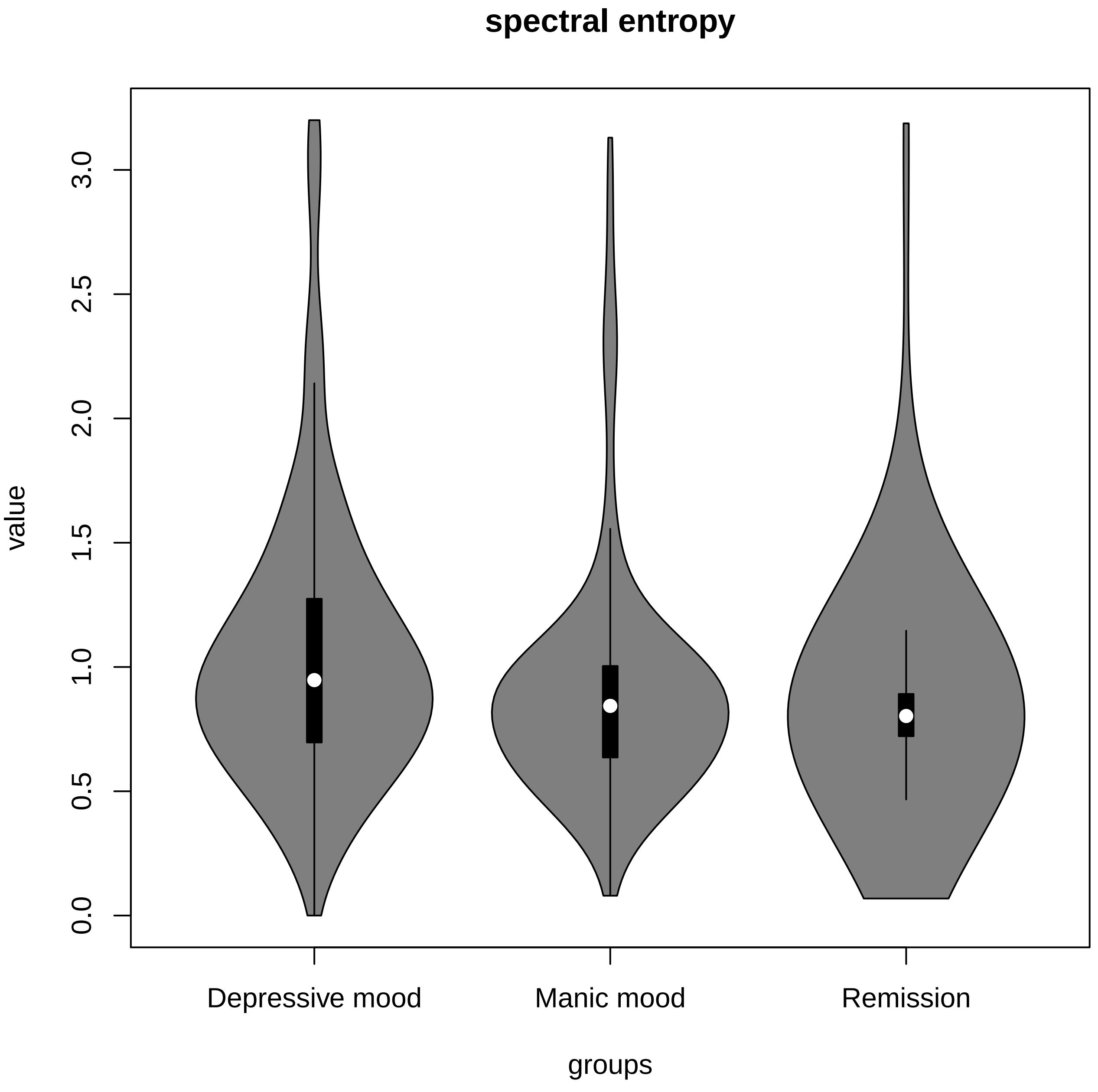
spectral entropy
value
0.0
0.5
1.0
1.5
2.0
2.5
3.0
Depressive mood
Manic mood
Remission
groups

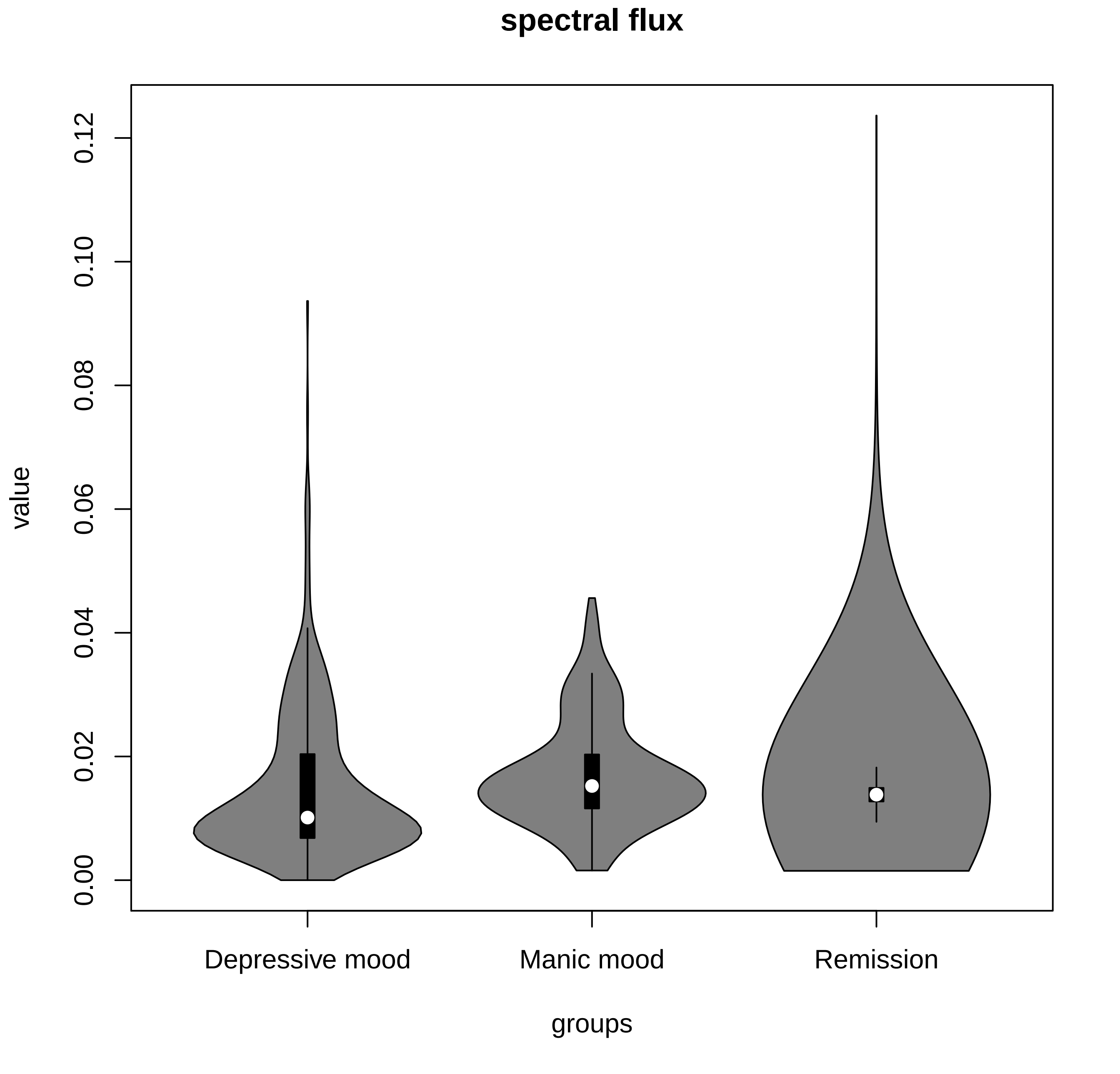
spectral flux
value
0.00
0.02
0.04
0.06
0.08
0.10
0.12
Depressive mood
Manic mood
Remission
groups

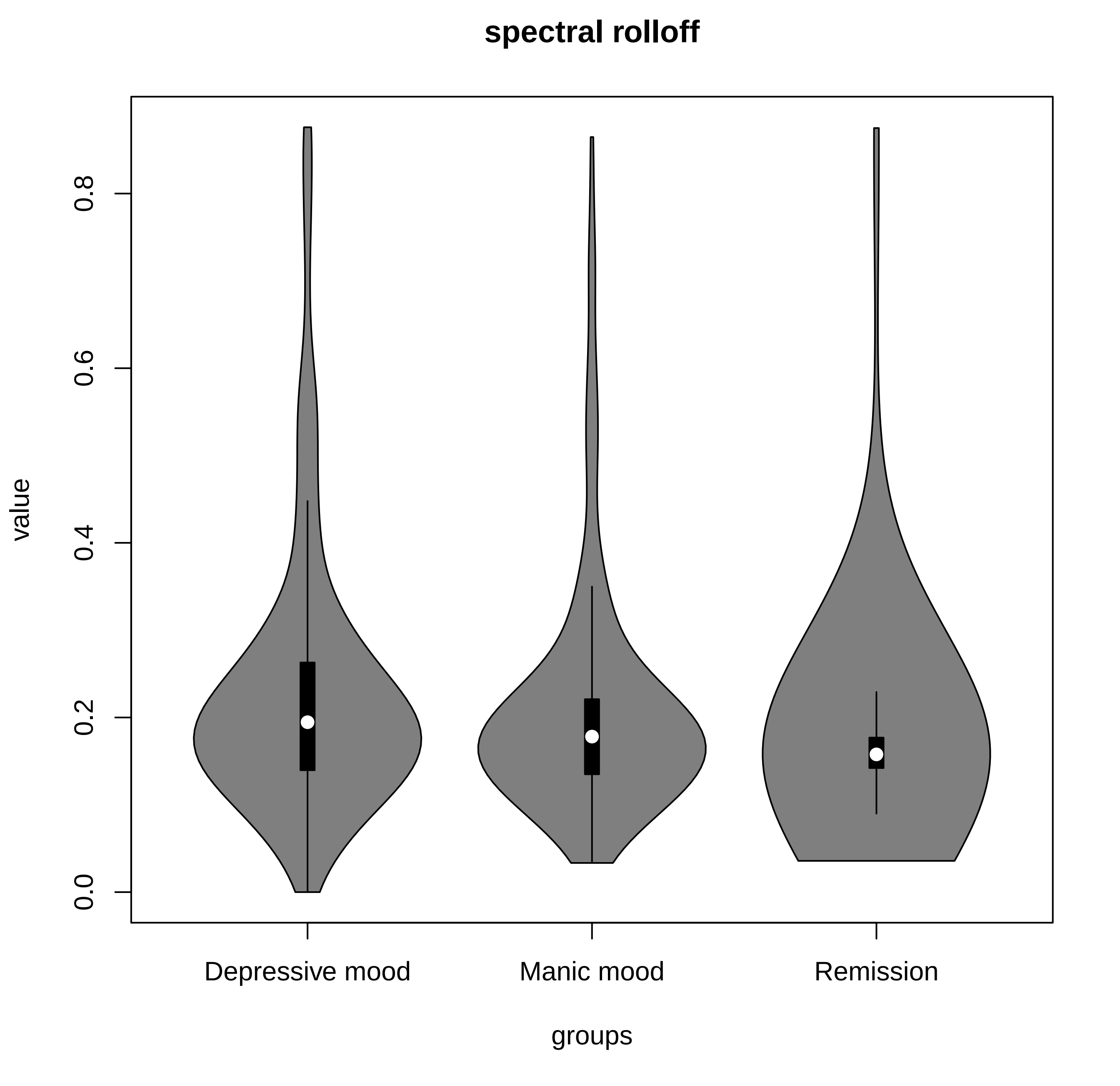

spectral rolloff
value
0.0
0.2
0.4
0.6
0.8
Depressive mood
Manic mood
Remission
groups

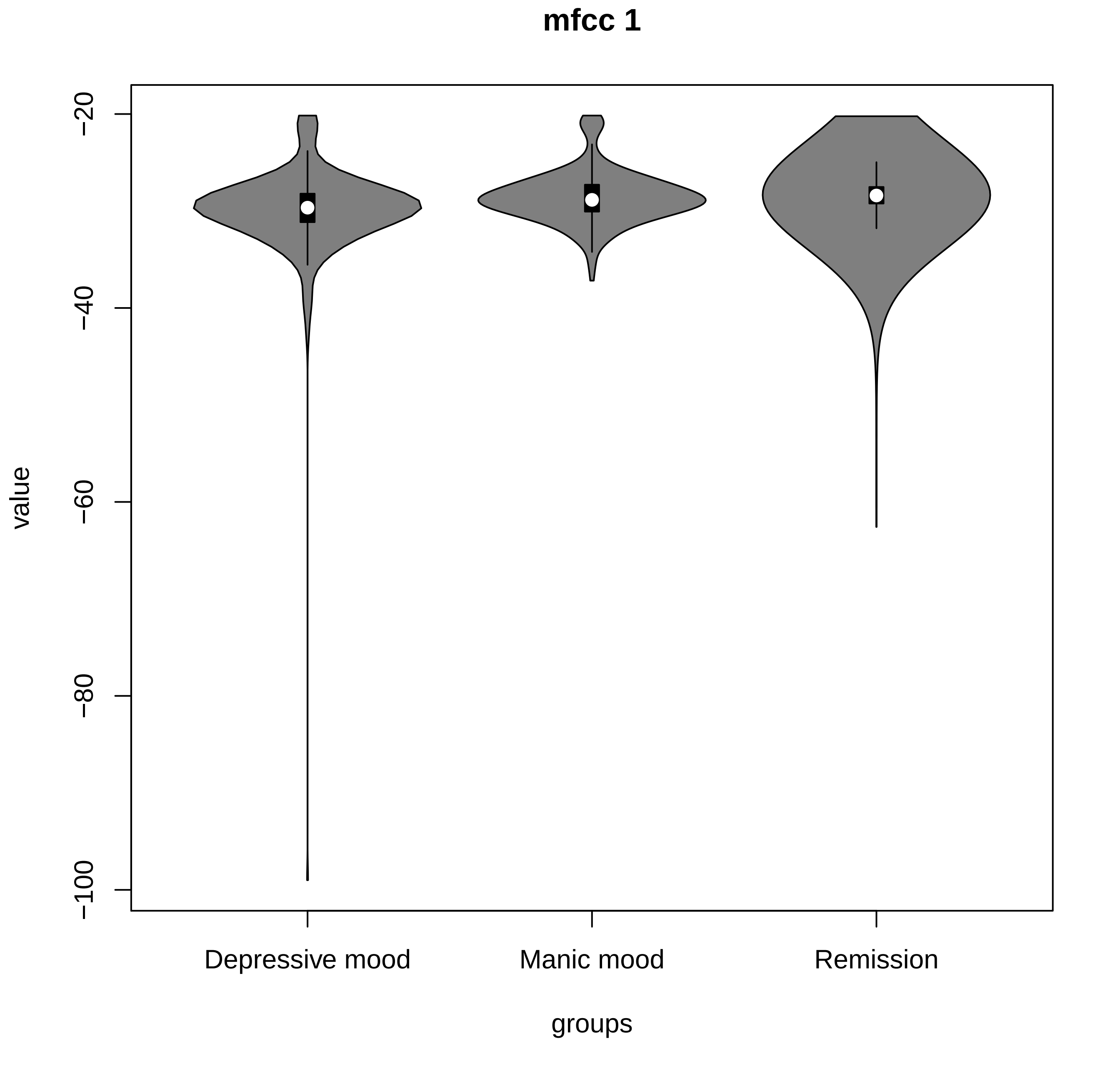

mfcc 1
−20
−40
−60
−80
−100
value
Depressive mood
Manic mood
Remission
groups

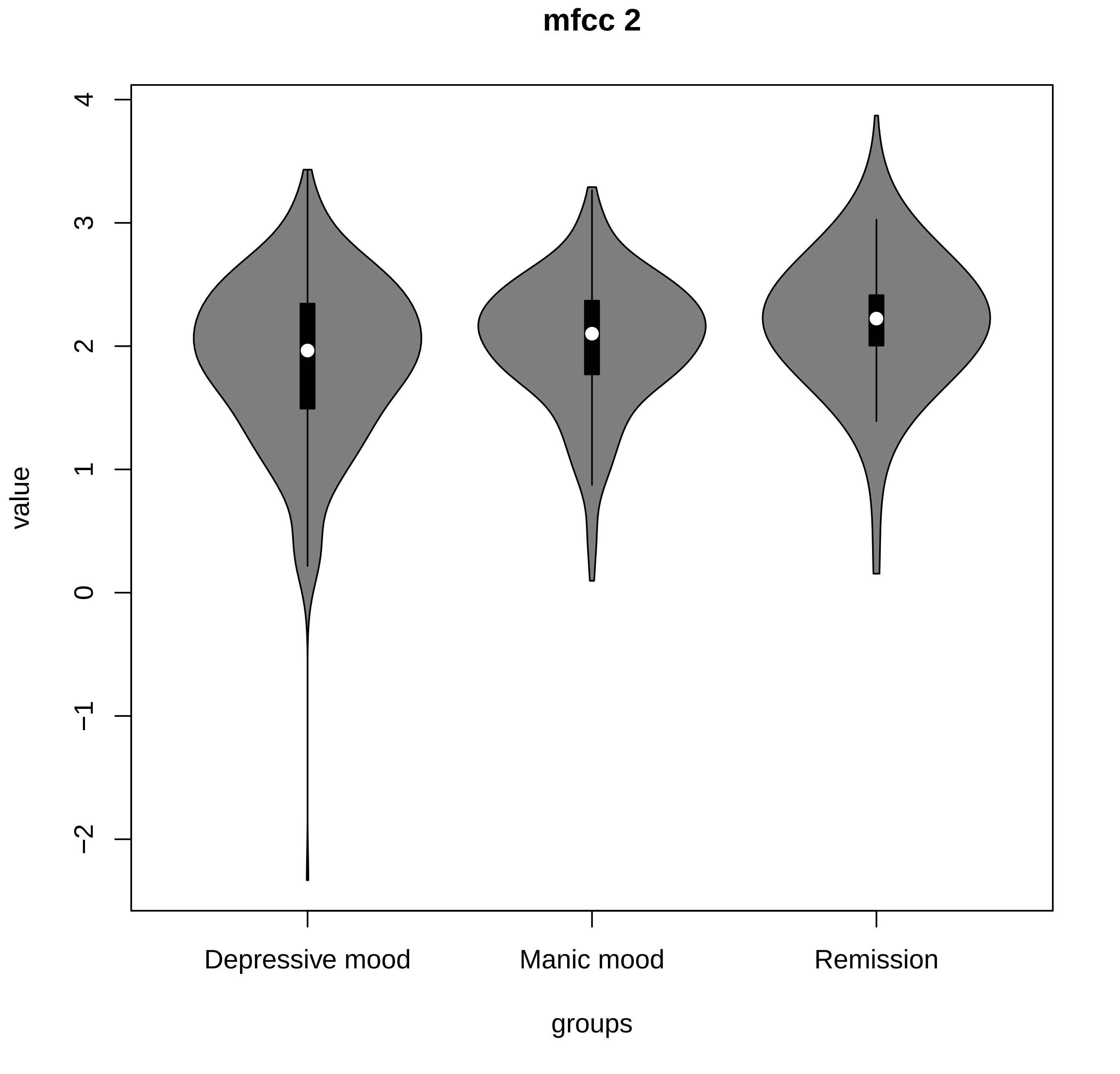
mfcc 2
value
4
3
2
1
0
−1
−2
Depressive mood
Manic mood
Remission
groups

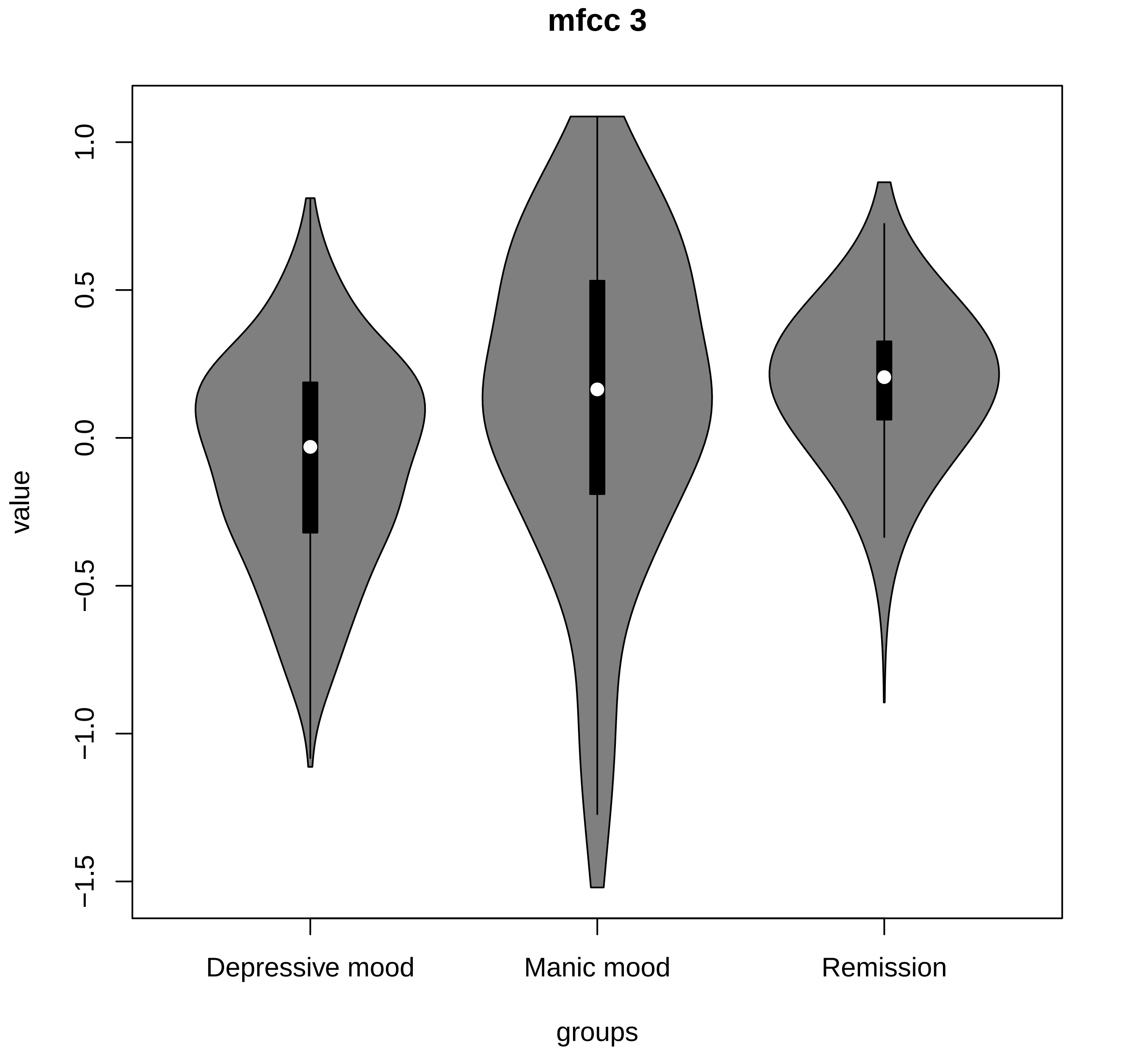

mfcc 3
value
1.0
0.5
0.0
−0.5
−1.0
−1.5
Depressive mood
Manic mood
Remission
groups

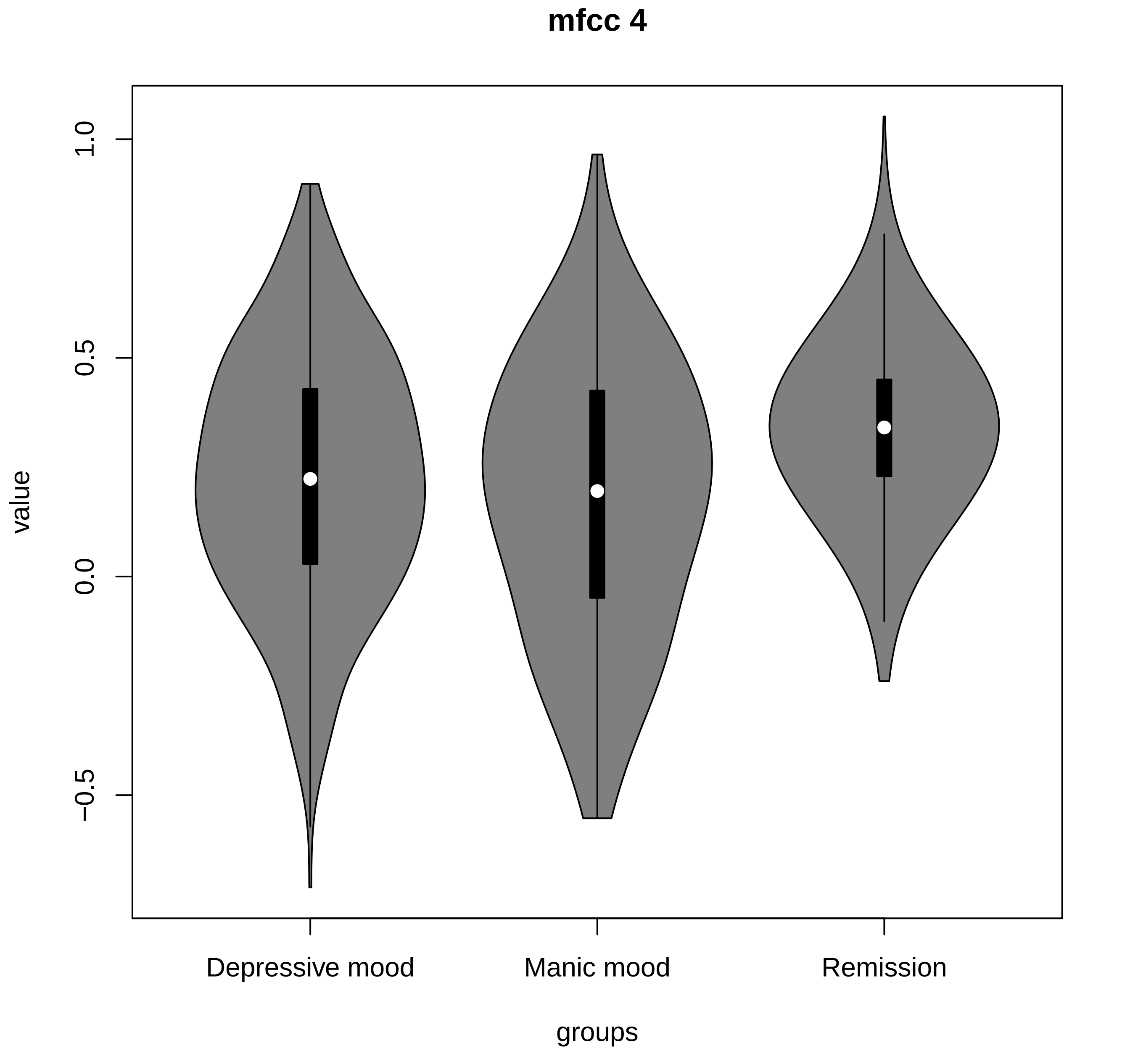
mfcc 4
value
1.0
0.5
0.0
−0.5
Depressive mood
Manic mood
Remission
groups

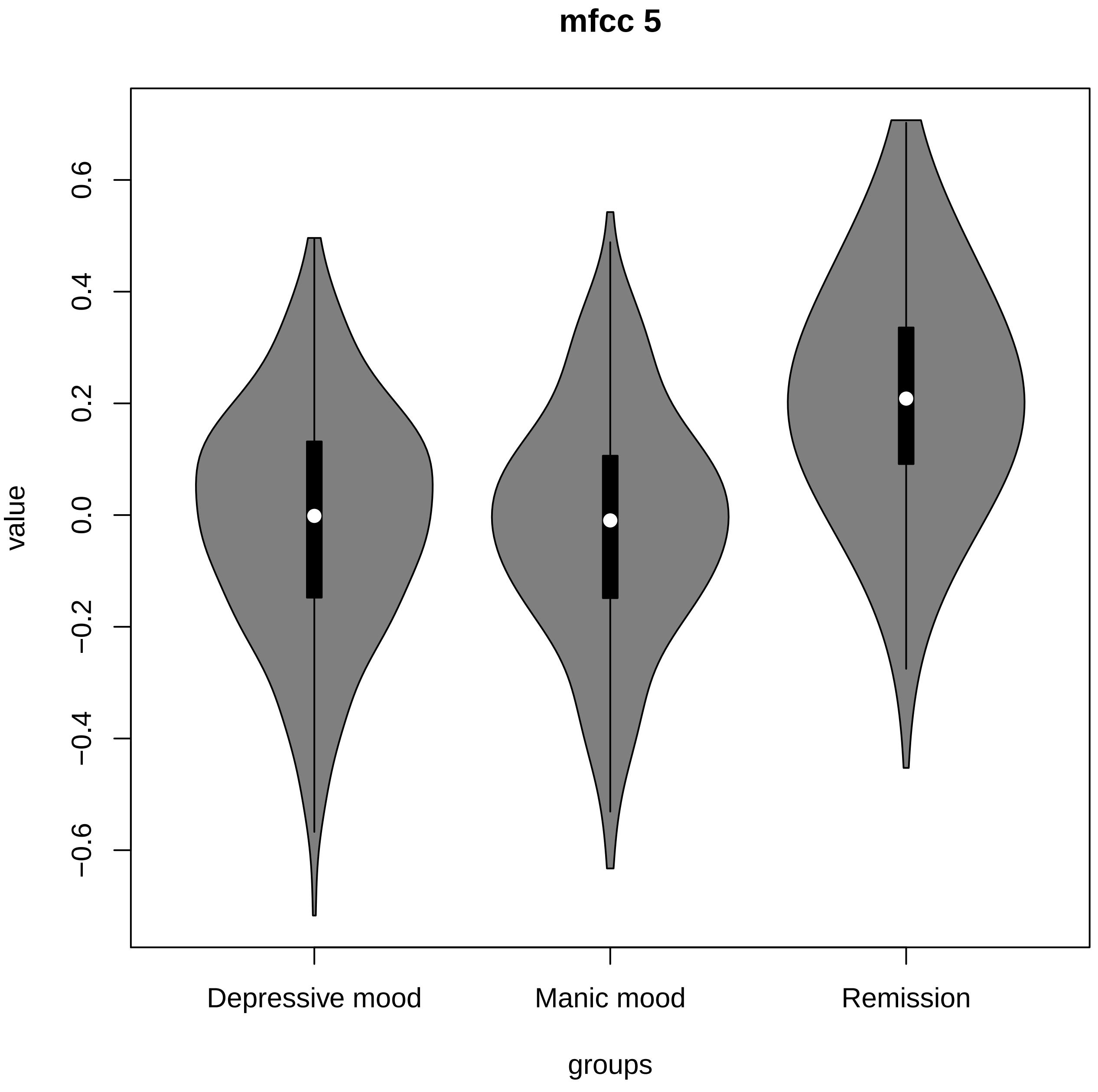
mfcc 5
value
0.6
0.4
0.2
0.0
−0.2
−0.4
−0.6
Depressive mood
Manic mood
Remission
groups

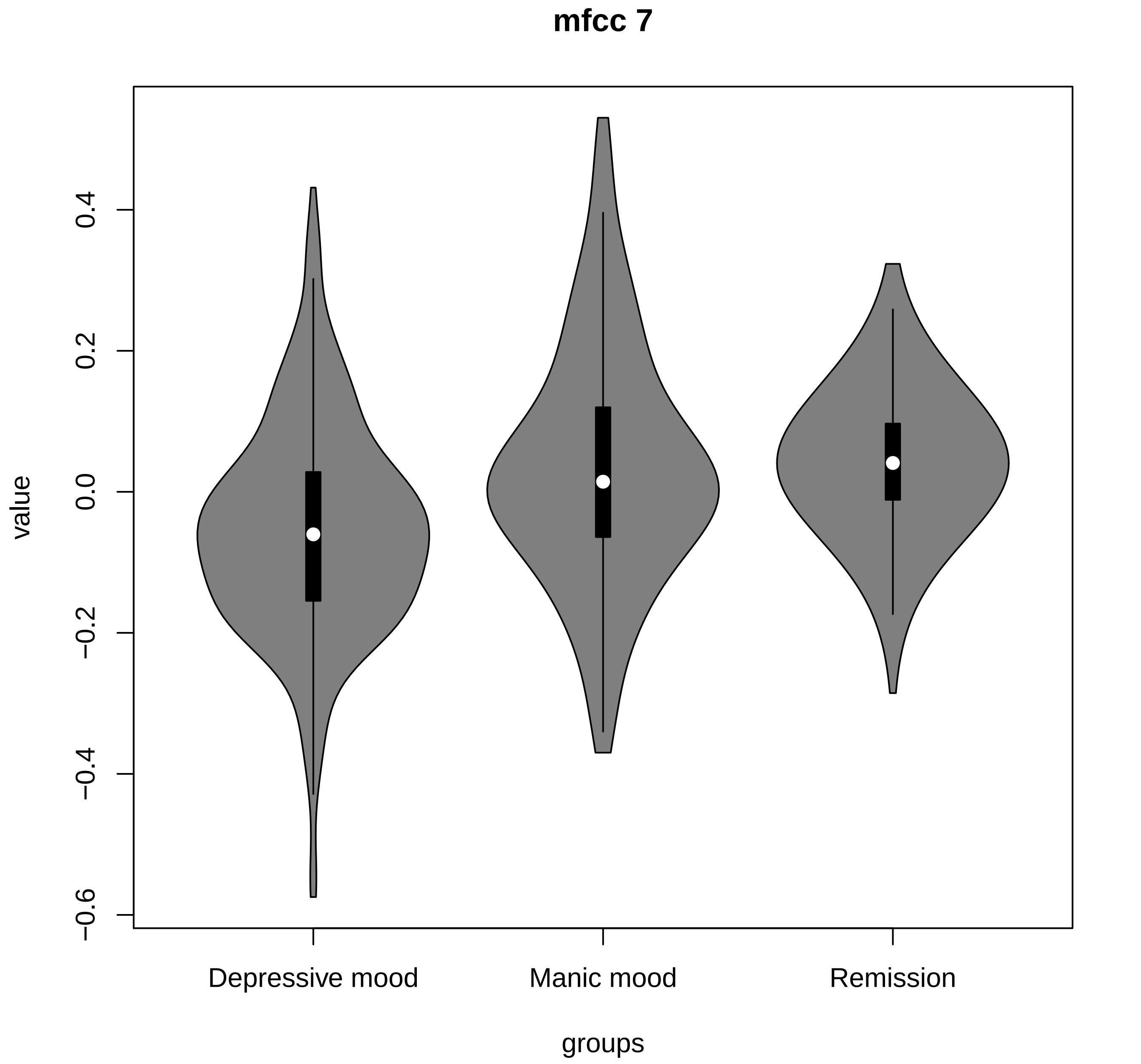

mfcc 7
value
0.4
0.2
0.0
−0.2
−0.4
−0.6
Depressive mood
Manic mood
Remission
groups

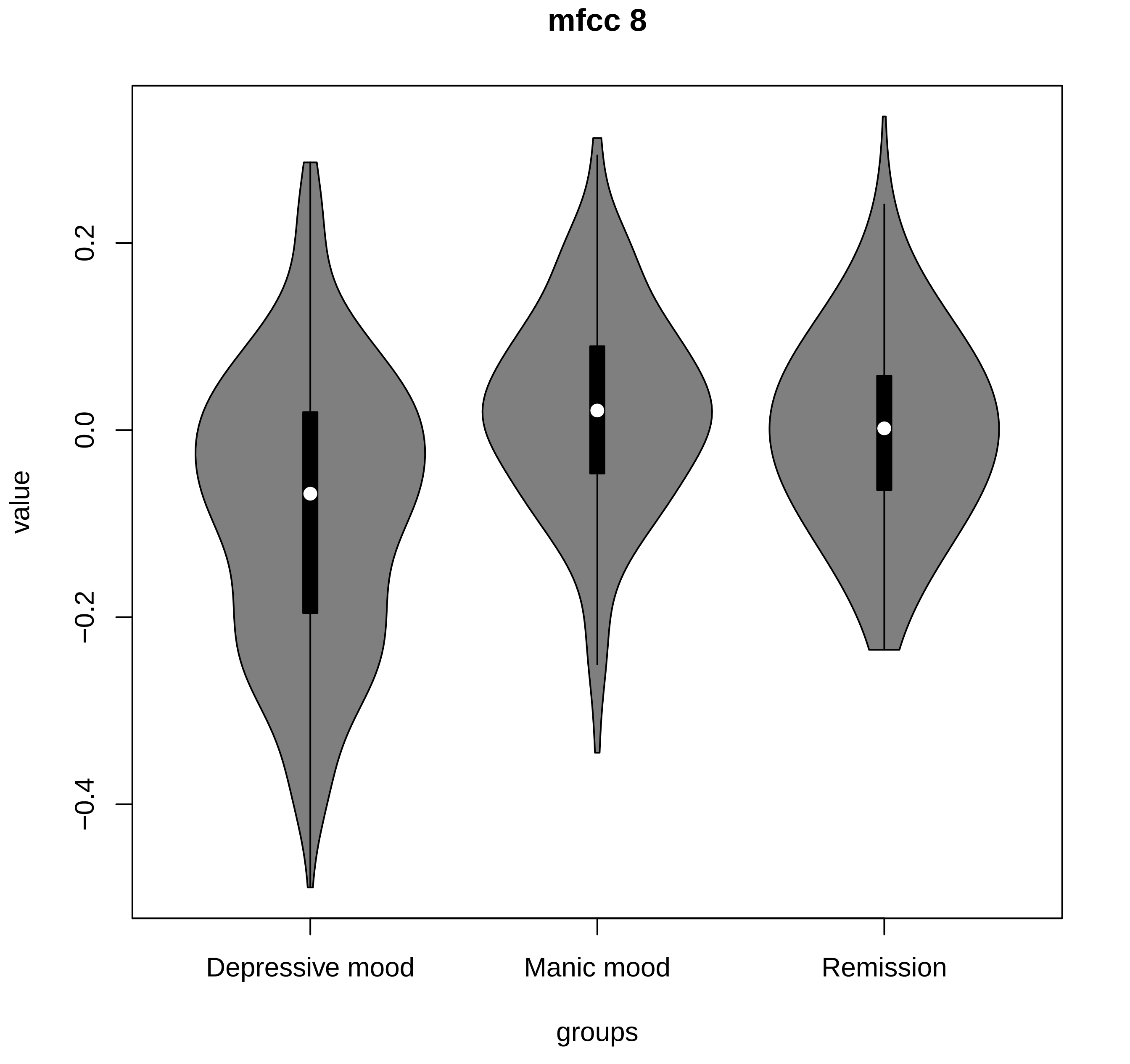
mfcc 8
value
0.2
0.0
−0.2
−0.4
Depressive mood
Manic mood
Remission
groups

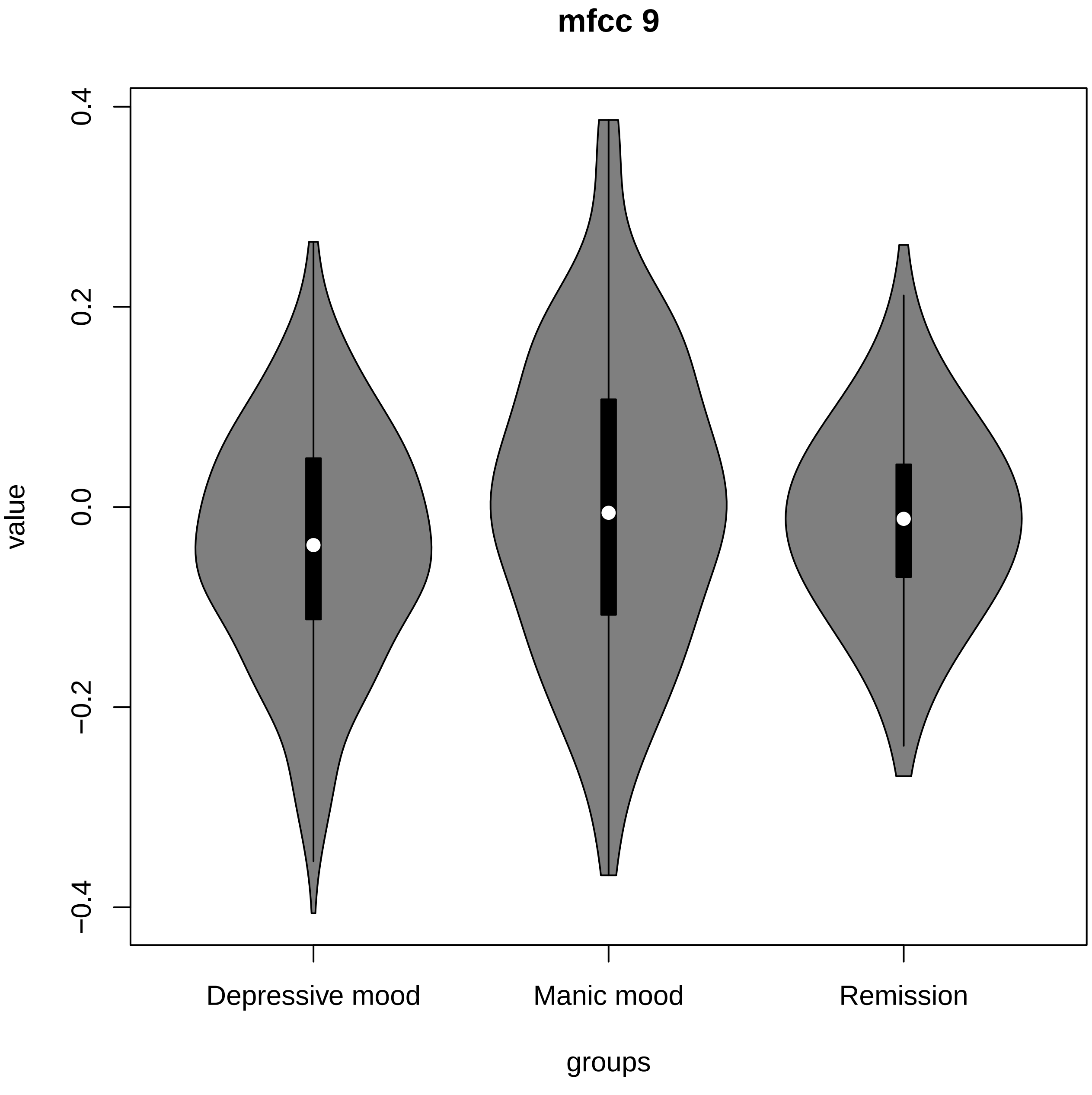
mfcc 9
value
0.4
0.2
0.0
−0.2
−0.4
Depressive mood
Manic mood
Remission
groups

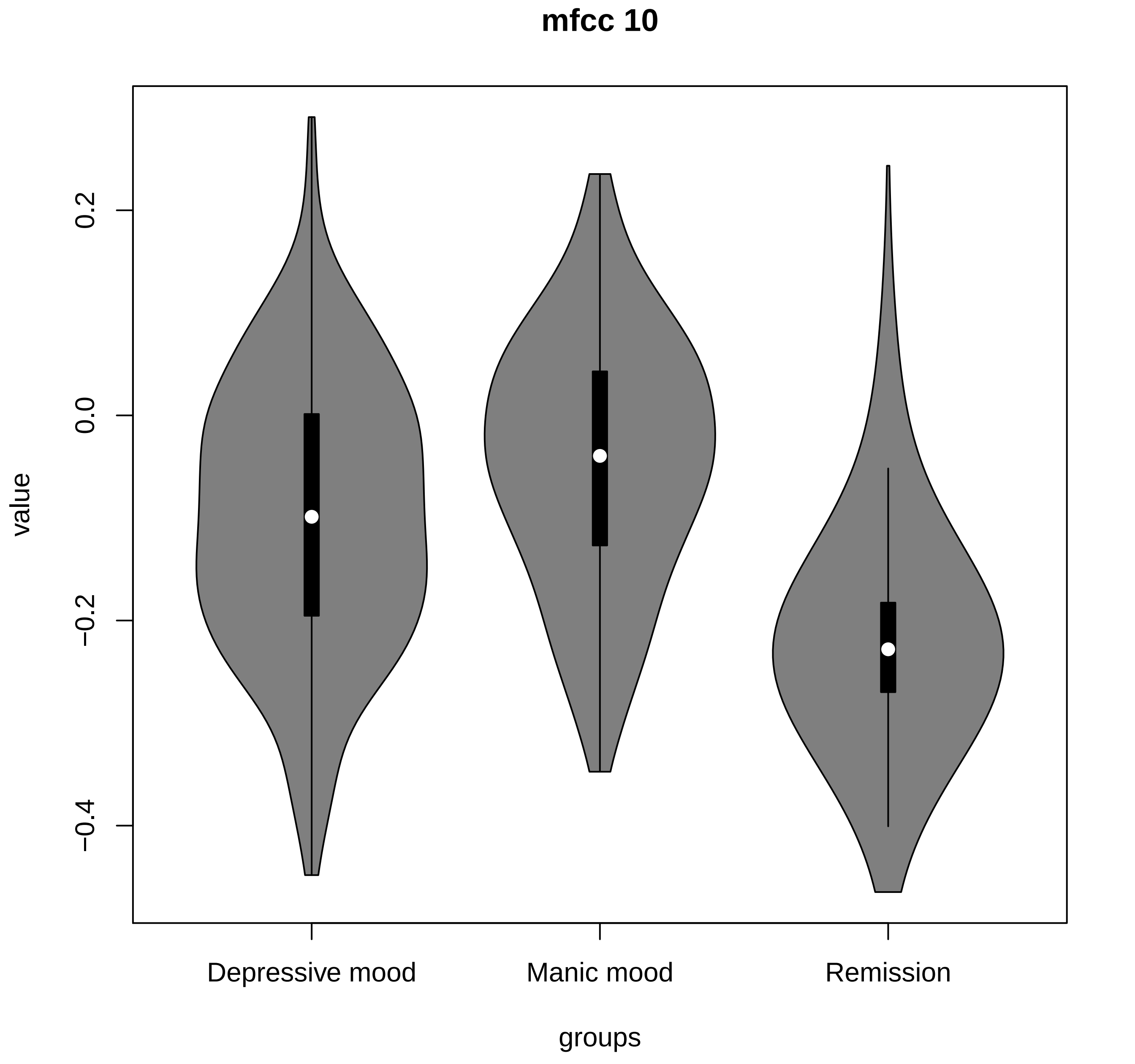
mfcc 10
0.2
0.0
−0.2
−0.4
value
Depressive mood
Manic mood
Remission
groups

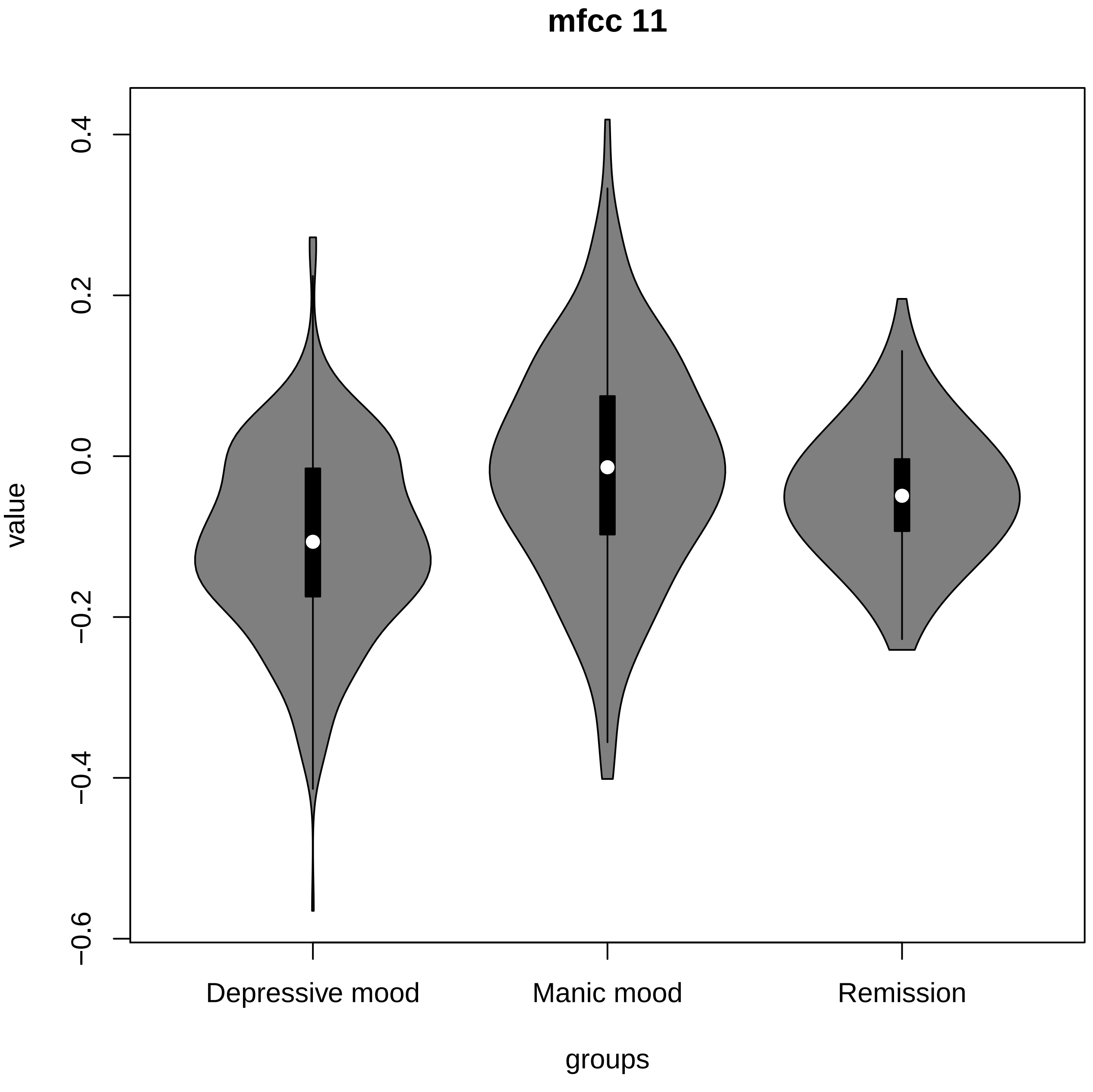
mfcc 11
value
groups
Depressive mood
Manic mood
Remission
0.4
0.2
0.0
−0.2
−0.4
−0.6

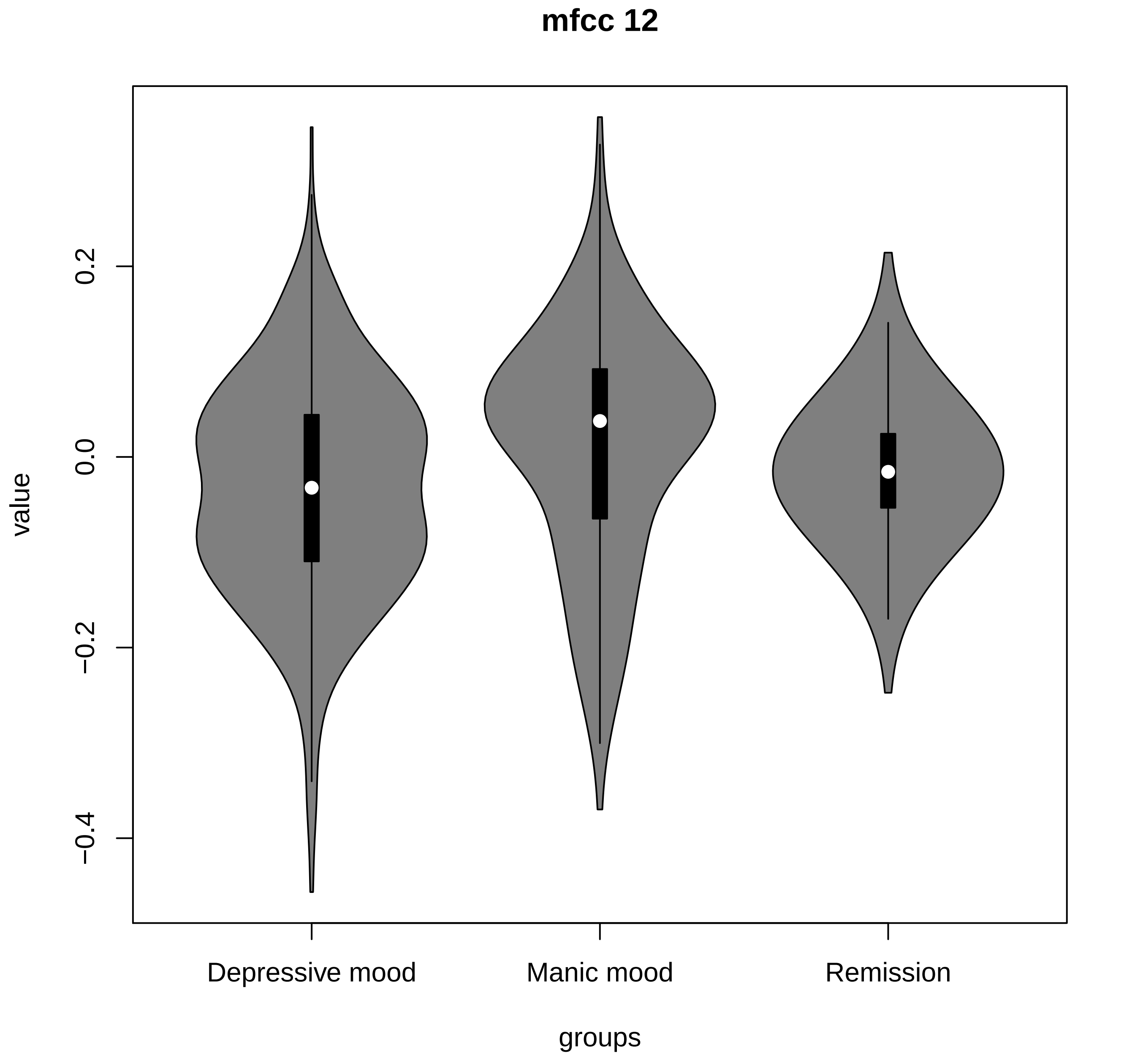
mfcc 12
value
0.2
0.0
−0.2
−0.4
Depressive mood
Manic mood
Remission
groups

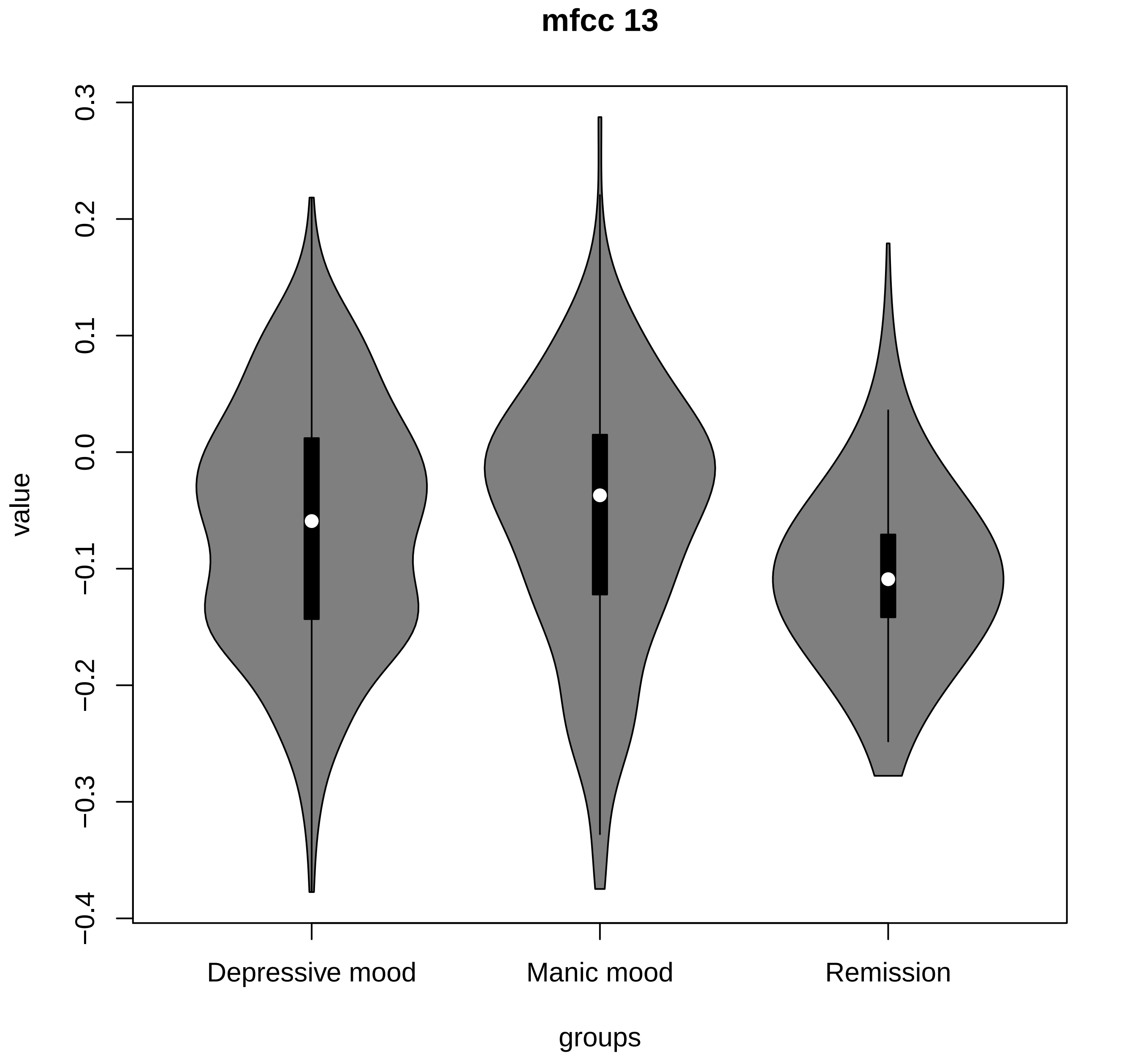
mfcc 13
value
0.3
0.2
0.1
0.0
−0.1
−0.2
−0.3
−0.4
Depressive mood
Manic mood
Remission
groups

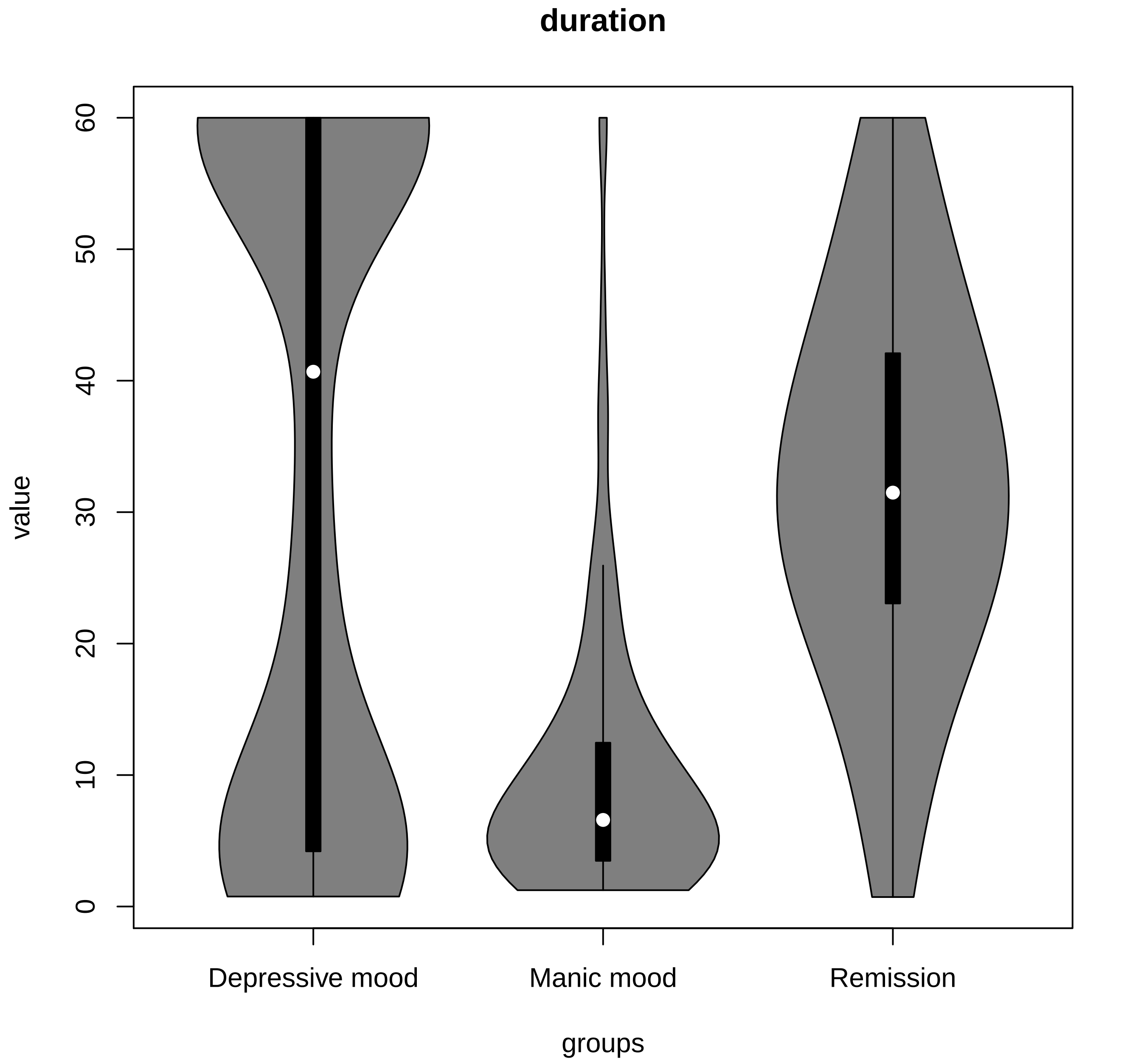
duration
value
0
10
20
30
40
50
60
Depressive mood
Manic mood
Remission
groups
